# Design, Synthesis and *In Vitro* Activity of Anticancer Styrylquinolines. The p53 Independent Mechanism of Action

**DOI:** 10.1371/journal.pone.0142678

**Published:** 2015-11-23

**Authors:** Anna Mrozek-Wilczkiewicz, Ewelina Spaczynska, Katarzyna Malarz, Wioleta Cieslik, Marzena Rams-Baron, Vladimír Kryštof, Robert Musiol

**Affiliations:** 1 A. Chełkowski Institute of Physics, University of Silesia, Katowice, Poland; 2 Silesian Center for Education and Interdisciplinary Research, University of Silesia, Chorzów, Poland; 3 Institute of Chemistry, University of Silesia, Katowice, Poland; 4 Laboratory of Growth Regulators, Centre of the Region Haná for Biotechnological and Agricultural Research, Palacký University and Institute of Experimental Botany AS CR, Olomouc, Czech Republic; Virginia Commonwealth University, UNITED STATES

## Abstract

A group of styrylquinolines were synthesized and tested for their anti-proliferative activity. Anti-proliferative activity was evaluated against the human colon carcinoma cell lines that had a normal expression of the p53 protein (HCT116 p53^+/+^) and mutants with a disabled TP53 gene (HCT116 p53^-/-^) and against the GM 07492 normal human fibroblast cell line. A SAR study revealed the importance of Cl and OH as substituents in the styryl moiety. Several of the compounds that were tested were found to have a marked anti-proliferative activity that was similar to or better than doxorubicin and were more active against the p53 null than the wild type cells. The cellular localization tests and caspase activity assays suggest a mechanism of action through the mitochondrial pathway of apoptosis in a p53-independent manner. The activity of the styrylquinoline compounds may be associated with their DNA intercalating ability.

## Introduction

**Fig 1:**
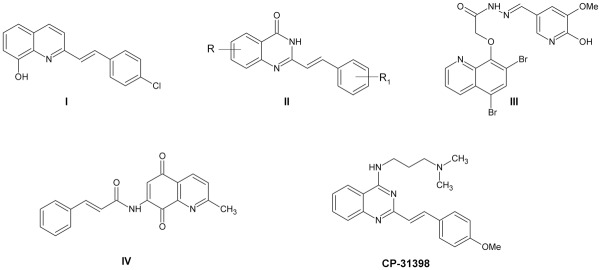
Quinolines and analogs with a marked anticancer activity.

Styrylquinolines are interesting quinoline-related compounds that have a broad spectrum of biological activity [1–7]. The anti-proliferative effect of quinoline-5,8-diones and styrylquinoline-carboxylic acids on tumor cell lines have been observed [8,9]. In this respect, compound **I** (Fig 1) demonstrated a marked anti-proliferative effect with IC_50_ = 0.77 μM (SK-N-MC cell line) [7]. More recently, we presented a similar series of compounds that also appeared to be active. Among them the analog of **II** (R = H, R_1_ = OH) shown activity at micromolar level IC_50_ = 1.5 μM (HCT116 p53^+/+^ cell line) [9]. Another very important group to be mentioned here are quinolinediones. This moiety is the main fragment of lavendamycin and related compounds, which are known for their broad spectrum of activity and a series of 7-amino-quinaldine-5,8-diones whose anti-proliferative activity has been reported [8,10–13]. Among these, compound **IV** appeared to be active (IC_50_ = 1.44 μM; P388 cell line) [8]. Jiang et al. synthesized a large series of quinazolines (**II**) in a search for new tubulin polymerization inhibitors [14] and some were found to be highly active against L1210 leukemia cells. 6-methoxyderivative (**II**, R = OMe, R_1_ = H) showed activity at micromolar concentrations (IC_50_ = 3.59 μM).

The styrylquinazoline CP-31398 is a small molecule with a unique ability to stabilize and activate the p53 protein even in some cell lines with mutant p53. The p53 is a tumor suppressor protein that is responsible for initiating growth arrest, DNA repair or apoptosis. It is induced by various endo- and exogenous factors such as DNA damage, radicals, radiation and thermal or chemical stress. Roughly 50% of all cancers have mutated the TP53 gene, which has been connected with drug resistance and a poor prognosis [15]. Structurally diverse agents from small organic molecules to oligopeptides that can reactivate the altered protein in TP53 mutated cells [16–18] are well known. Their activity opens fascinating new possibilities in anticancer treatment. The compound CP-31398 has been broadly studied for its potency in restoring the mutant p53 functions, e.g., its sequence-specific binding ability to DNA [19–23]. However, it has also been reported that TP53 may be upregulated in response to CP-31398 treatment [24]. Concluding, styrylquinazoline CP-31398 is cytotoxic against wild-type or mutant cells but not in cells where TP53 is deleted (p53-null or p53^-/-^) due to its specific mechanism of action [24,25]. As was shown by Luu and coworkers, this compound is several times less active against HCT116 p53^-/-^ cells than against their wild-type (p53^+/+^) counterparts [26]. A minute activity in null cells has been suggested to rely on the formation of ROS or activation of another proteins as p63 or p73 [24,27]. The last suggestion should, however, be rejected after the verifying report of Demma et al [22].

## Materials and Methods

All of the reagents were purchased from Sigma Aldrich. A Kieselgel 60, 0.040–0.063 mm (Merck, Darmstadt, Germany) was used for the column chromatography. TLC experiments were performed on alumina-backed silica gel 40 F254 plates (Merck, Darmstadt, Germany). The plates were illuminated under UV (254 nm) and evaluated in iodine vapor. The melting points were determined on an Optimelt MPA-100 apparatus (SRS, Stanford CA). The purity of the final compounds was determined using HPLC. Detection wavelengths of 210 and 250 nm were chosen for detection. The purity of individual compounds was determined from the area peaks in the chromatogram of the sample solution in order to ensure >95% purity. UV spectra (λ, nm) were determined on a Waters Photodiode Array Detector 2996 (Waters Corp., Milford, MA, U.S.A.) in a methanolic solution (*ca*. 6×10^-4^mol) and log ε (the logarithm of molar absorption coefficient, ε) was calculated for the absolute maximum λ_max_ of the individual target compounds. All NMR spectra were recorded on a Bruker AM-400 (399.95 MHz for ^1^H and 100 MHz for ^13^C), BrukerBioSpin Corp., Germany. Chemical shifts are reported in ppm (δ) against the internal standard, Si(CH_3_)_4_. Easily exchangeable signals were omitted when they were diffuse. Signals are designated as follows: s, singlet; d, doublet; dd, doublet of doublets; t, triplet; m, multiplet; bs, broad singlet. The detailed information on all compounds are presented in S1 File.

### Synthesis

The designed styrylquinolines were prepared from quinaldines in acetic anhydride according to a general procedure.

#### General method A

The appropriate quinoline derivative (2.5 mmol) in acetic anhydride was thoroughly mixed with two equiv aldehyde and heated in an inert gas atmosphere (N_2_) for 16 h at 130°C. Then, the mixture was evaporated to dryness and a solid was crystallized from EtOH.

Hydrolysis of acetoxy groups.

#### General method B

The crude product from step A was transferred to a mixture of pyridine and water at a ratio of 3:1 and further heated for 3 h at 100°C. Then, the mixture was evaporated to dryness and a solid was crystallized or chromatographed.

#### General method C

The appropriate styrylquinoline derivative (2.5 mmol) in methanol was thoroughly mixed with 2.5 equiv K_2_CO_3_ for 2 h at room temperature. Then, concentrated HCl was added and the resulting precipitate was filtered and washed with H_2_O. The crude product was crystallized from EtOH.

### UV-VIS spectroscopy

The absorption and fluorescence spectra were determined using U-2900 and F-7000 spectrometers (Hitachi), respectively. Measurements were performed at room temperature in a 10-mm quartz cell with spectroscopic grade dimethyl sulfoxide (DMSO) applied as a solvent. The most important parameters that were established from registered spectra are summarized in S1 Table.

### Cell lines

The human colon adenocarcinoma cell line HCT116 along with wild type p53 (p53^+/+^) were obtained from the ATCC. The normal human fibroblast cell line GM 07492 and HCT116 with a p53 deletion (p53^-/-^) were kindly provided by prof. M. Rusin from Maria Sklodowska-Curie Memorial Cancer Center and Institute of Oncology in Gliwice, Poland. Cells were grown as monolayer cultures in 75 cm^2^ flasks (Nunc) in Dulbecco’s modified Eagle’s medium with an antibiotic gentamicin (200 μL/100 mL medium). DMEM for HCT116 were supplemented with 12% heat-inactivated fetal bovine serum (Gibco) and for GM 07492 with 15% fetal bovine serum (Gibco). Cells were cultured under standard conditions at 37°C in a humidified atmosphere at 5% CO_2_.

### Biological activity measurements

#### MTS assay

The cells were seeded in 96-well plates 24 h before the addition of the compounds that were tested. The assay was performed following a 72 h incubation with varying concentrations of the agents that were tested. The results were calculated as IC_50_ values (using GraphPad Prism 5). Each individual compound was tested in triplicate in a single experiment with each experiment being repeated three times. After a 72 h incubation with the compounds that were tested, 20 μL of The CellTiter 96^®^AQueous One Solution—MTS (Promega) solution was added to each well (with 100 μL DMEM without phenol red) and incubated for 1 h at 37°C. The optical densities of the samples were analyzed at 490 nm. Results were expressed as a percentage of the control. The inhibitory concentration (IC_50_) was defined as the compound concentration that was necessary to reduce the proliferation of cells to 50% of the untreated control. The results are summarized in Table 1.

**Table 1:** Anti-cancer activity of the studied compounds (ND—not determined).

Compounds		Activity IC_50_ [μM]		
No	R_1_/R_2_	HCT116 (p53^+/+^)	HCT116 (p53^-/-^)	GM 07492
1a	-/2-OH	17.45±2.20	14.05±2.24	>25
2a	-/4-OEt	>25	>25	ND
3a	-/4-OBu	>25	>25	ND
1b	H/3,5-OMe	>25	>25	ND
2b	Ac/2-OAc	**8.08±0.56**	14.71±1.74	6.92±1.72
3b	H/2-OAc	**7.32±1.01**	13.53±0.81	>25
4b	H/3-OAc	**7.88±1.98**	10.73±3.76	>25
5b	H/2,3-Cl	**5.13±1.41**	**2.99±0.61**	>25
6b	H/3,4-Cl	**9.41±2.17**	**3.34±0.58**	>25
7b	H/3,4-OAc,5-OMe	>25	>25	ND
8b	Ac/2,4-OAc	>25	>25	ND
9b	H/2,4,6-OH	>25	>25	ND
10b	Ac/2,4,6-OAc	>25	>25	ND
11b	H/2-F	15.43±2.46	**8.38±1.12**	>25
12b	Ac/2-F	12.07±3.02	**5.78±0.78**	>25
13b	H/3-OMe	24.60±4.36	18.42±1.36	>25
14b	Ac/2,3,4-OAc	17.88±1.67	11.36±1.02	ND
15b	H/2-OEt	15.81±3.32	12.80±3.53	ND
16b	H/4-OEt	>25	16.11±4.84	ND
17b	H/2-Cl	**9.84±1.79**	**7.97±3.24**	20.17±6.52
18b	H/2-OH	16.07±0.88	15.25±1.31	ND
19b	H/3-OH	16,31±0,76	10.62±1.23	ND
20b	H/2,4-OH	>25	>25	ND
1c	-/2-OEt	**5.93±0.97**	**3.25±1.81**	>25
2c	-/2-Cl	**1.88±0.85**	**2.86±1.00**	12.57±2.88
3c	-/4-OEt	**2,23±0.81**	**3.53±0.83**	>25
Doxorubicin		5.95±0.5	1.65±0.21	3.38±1.29
CP-31398		18.63±0.92	26.28±1.41	12.26±0.54

Results are expressed as mean ± SD of at least three independent experiments. IC_50_ values below 10 μM are bolded.

#### Subcellular localization

Compound localization in living cell cultures was studied using fluorescence microscopy. Cells were seeded at a density of 20,000 cells/well on 8-well Lab-Tek chambered coverglasses (Nunc). After 18 h, a medium containing the compounds at a concentration that inhibits 50% viability of cells was added for an additional 2 h (standard conditions). Then the cells were rinsed with PBS (pH 7.2) and a serum-free medium that contained MitoTracker^®^ Orange CMTMRos dye (100 nM, 30-min incubation, Molecular Probes) was added. After incubation, the cells were washed twice with PBS and maintained in a phenol-free medium. Image observation was carried out immediately after staining using a confocal LSM700 system (Carl Zeiss). Additionally, an inverted fluorescence microscope (IX81, Olympus) equipped with a CO_2_ incubator (temperature, humidity and gas flow under control) was used to confirm the mitochondrial localization of compounds that was tested. In addition to the MitoTracker^®^ Orange CMTMRos dye, LysoTracker^®^Yellow-HCK-123 dye (5 μM, 1 h incubation, Molecular Probes) was applied.

#### Caspase activity assay

To measure caspase-3 and -7 activities, a luminescent Caspase-Glo 3/7 assay was performed. HCT116 (p53^+/+^) and (p53^-/-^) cells were seeded onto black 96-well plates at a density of 3,500 cells/well. After 24 h the compounds that were tested (**2c, 3c, 3b, 4b, 5b, 6b, 13b, CP-31398**) and a reference (doxorubicin) at IC_50_ concentration were added. After 48 h a Caspase-Glo 3/7 Assay (Promega) was performed according to the manufacturer’s instructions. After adding 100 μL of Caspase3/7 GloReagent, cells were incubated for 2.5 h at room temperature. The luminescence was measured using a multi-plate reader (Synergy 4, Bio Tek) with an integration time of 1 second per well. The experiments were performed in duplicate.

#### Immunoblotting

Specific antibodies were purchased from Sigma-Aldrich (anti-PARP, anti-tubulin and peroxidase-labeled secondary antibodies). Briefly, cellular lysates were prepared by harvesting cells in a Laemmli sample buffer. Proteins were separated on SDS-polyacrylamide gels and electroblotted onto nitrocellulose membranes. After blocking, the membranes were incubated with specific primary antibodies overnight, washed and then incubated with peroxidase-conjugated secondary antibodies. Finally, peroxidase activity was detected with ECL+ reagents (AP Biotech) using a CCD camera LAS-4000 (Fujifilm).

#### Intercalation

For the DNA binding studies, Calf-thymus DNA (CT-DNA) was purchased from Sigma Aldrich. The lyophilized CT-DNA was dissolved in 10 mM Tris-HCl, pH 7.9, mixed gently and left overnight at 4°C. Then, the concentration of CT-DNA was determined from the absorbance at 260 nm using an extinction coefficient of 6600 M^-1^cm^-1^. Compounds **2c, 3c, 5b, 6b, CP-31398** and **doxorubicin** were dissolved in DMSO to a concentration of 8.35 mM, which were then used as the stock solution for the preparation of the various concentrations (25 μM; 12,5 μM; 6 μM) in 1 mL in 10 mM Tris-HCl (pH 7.9). Afterwards, 18 μM CT-DNA was added to the prepared solutions and were incubated for 1.5 h at 37°C with occasional vortexing. Absorption spectra were measured using a Hitachi U-2900 spectrophotometer in range of 200–600 nm. All absorption spectra were imported and compared in OriginPro 8.0. Results are shown in S3 Table.

#### Statistical analysis

The analysis of cell viability was performed using GraphPad Prism v.5.0 software (GraphPad Software, USA). The normality of variable distribution was assessed using the Shapiro-Wilk test. The non-parametric Mann-Whitney test was applied to detect significant differences between mean values.

## Results and Discussion

### Design and synthesis

**Fig 2:**
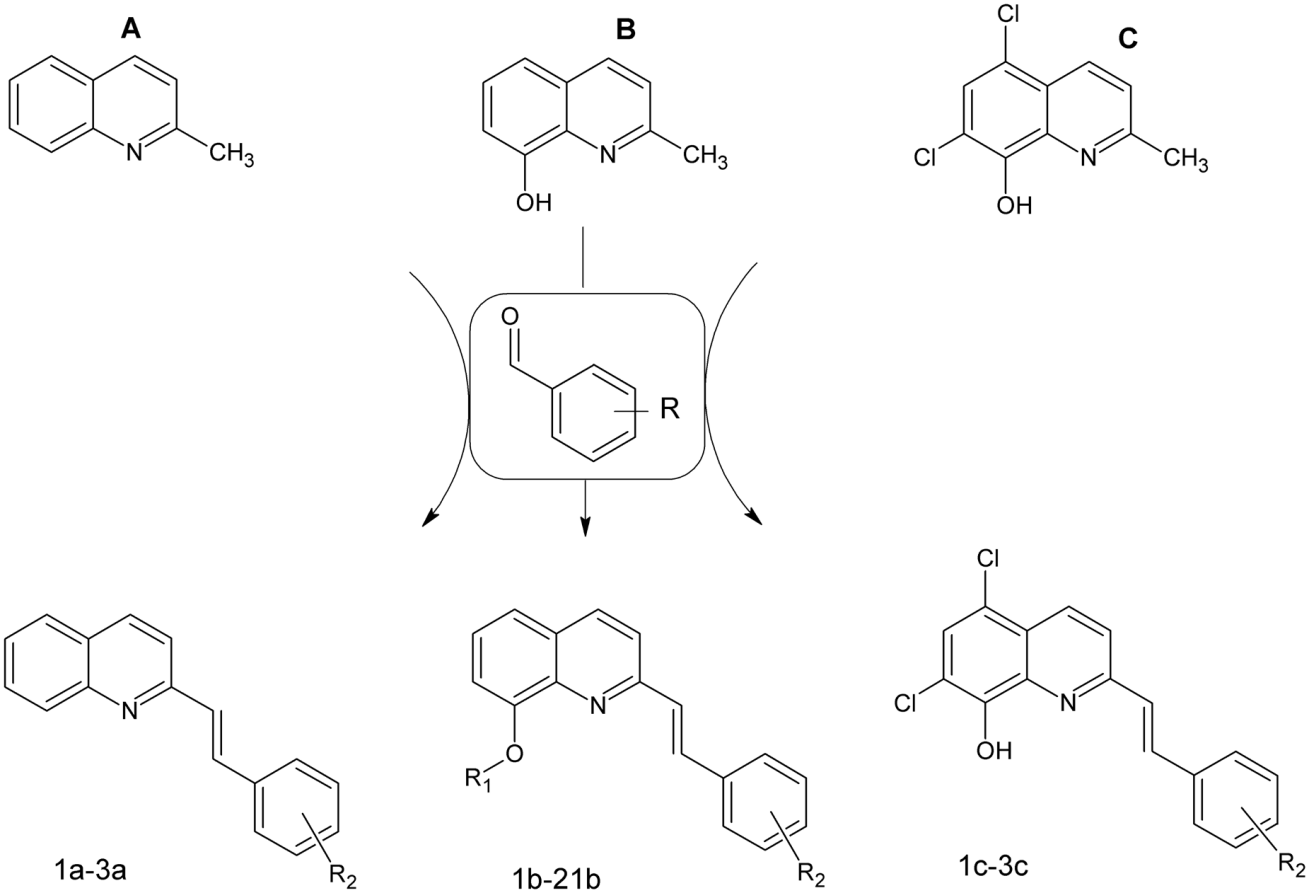
Synthesis of the compounds that were studied.

Based on our experience in the design and synthesis of biologically active stryrylquinolines and their structural similarity to CP-31398, we decided to screen the series of compounds on human colon cancer lines, wild-type and p53 negative. The results were, however, unexpected and lead to some interesting remarks. The compounds that were used in this study were obtained during our search for active styrylquinolines as published recently [2,7,28–33]. As this experiment was concentrated on the styryl part of the molecule, we modified it according to the idea that is depicted in Fig 2. Series of nine new compounds were obtained to fill the gap in the library of structures. Furthermore, we decided to evaluate dihalogenated compounds based on the 5,7-dichloroquinoline scaffold. The rationale for this was our recent findings on their potency [2]. Such a scaffold was also reported in the work of Arafa et al. in which structure **III** with IC_50_ = 4.7 μM was found to be promising against colon cancer cells. The importance of C-8 OH substitution was also studied here on the basis of the recent report of Chan et al. [34].

**Fig 3:**
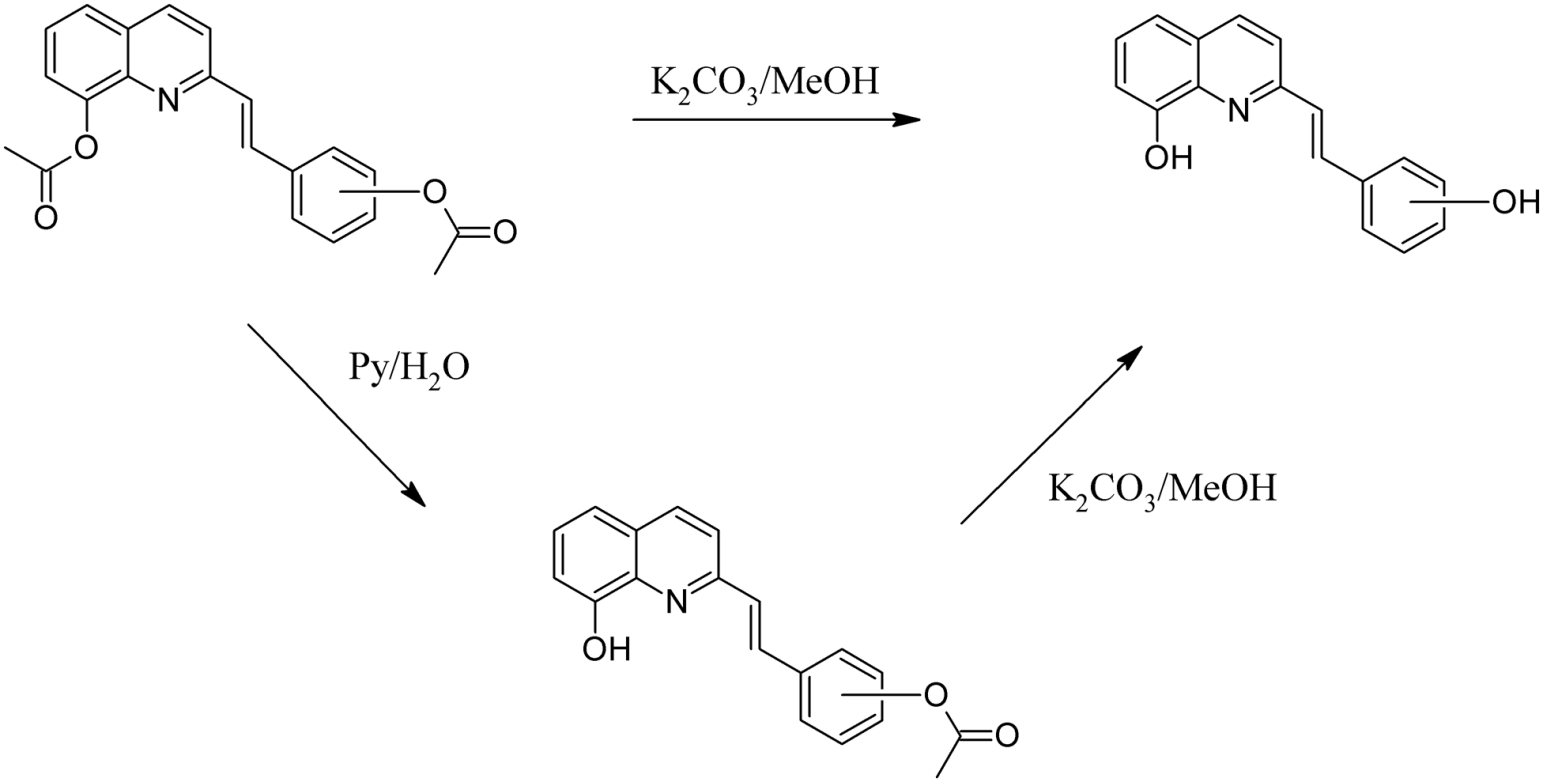
Selective hydrolysis of styrylquinolines. For example, compare **2b**, **3b** and **18b**.

The designed compounds were synthesized from the commercially available quinaldines: **A**, **B** and **C**. The first step of this synthesis consisted of condensation with the appropriate aldehyde in acetic anhydride in reflux conditions. In this solvent, all of the hydroxyl groups that were present in the substrates were acetylated. These groups were selectively removed with pyridine/water or K_2_CO_3_/methanol mixtures (Fig 3).

### Biological studies

The anti-proliferative activity of the synthesized compounds was tested with the MTS assay against the human colon carcinoma (HCT116) cell lines, wild-type and p53 negative. Additionally, the compounds were also tested for their cytotoxicity against normal cells—human fibroblasts (GM 07492). The results from the anti-proliferative activity assays are shown in Table 1 and S2 Table. All of the compounds could be divided into three groups (**A**, **B**, **C**) according to their structural scaffolds (Fig 2). The biological activity results are in agreement with this distribution. In general, compounds from group **A** can be regarded as inactive, while those from group **C** are the most active. Group **B** is more extensive because of its substituents and the activity spectrum. C-8 unsubstituted quinoline does not induce activity. 8-hydroxyquinoline or its dichloro-analog is a good scaffold for active structures. The acetylation of 8-OH apparently has no effect on activity at least within group **B** (compare **2b-3b** or **11b-12b**). The styryl part of the molecule should be substituted with halogen. Interestingly, an additional chlorine atom enhanced the activity (**5b** vs **17b**). The acetoxy group in the phenyl ring also had a positive effect on activity; however, a C-4 (**15b** vs **16b**) substitution as well as multisubstituted patterns (**3b**-**5b** vs **8b**, **10b**) decreased activity. The majority of the compounds that were tested were non-toxic to nonmalignant cells within the concentrations that were tested.

**Fig 4:**
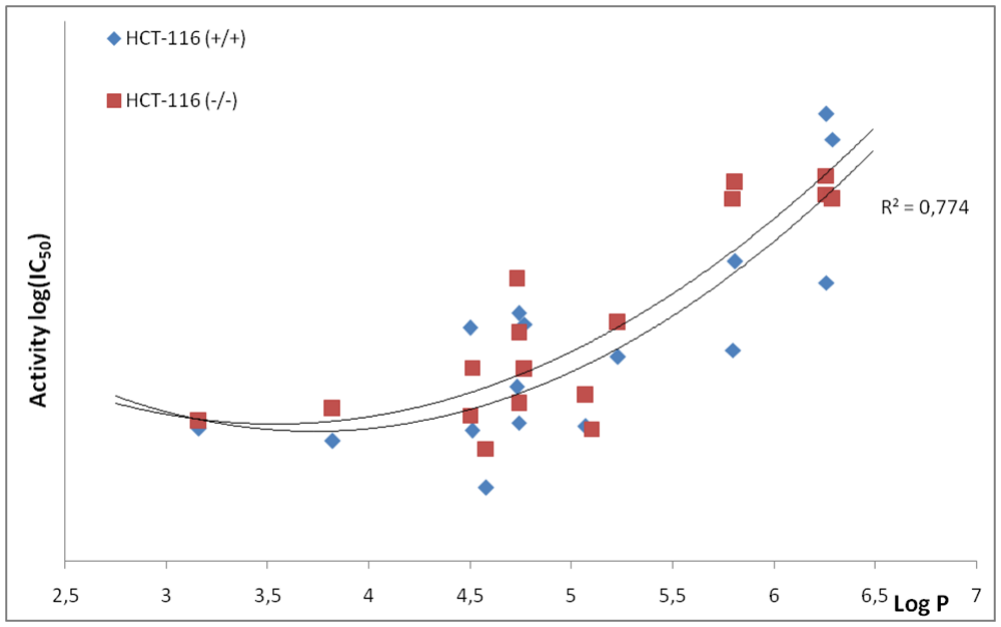
Activity (as—logIC_50_) vs logP of the compounds that were tested.

Some relationships with their lipophilicity could be drawn for the active compounds. In Fig 4, we present the active compounds (logIC_50_) plotted against their lipophilicity. All of the compounds with a similar potency to standard doxorubicin have LogP above 4.5. The more lipophilic the compounds were, the higher their antiproliferative activity. However, the shape of dependence may be astonishing. In general, according to the literature, a plateau should be observed at higher LogP values or even a decrease in the activity in the Gaussian shape function [35,36]. This, however, can be explained. First of all, in Fig 4, we plotted only the active compounds and therefore the range of lipophilicity is relatively narrow. However, the action of these compounds may be revealing in membranes or other more lipophilic structures and thus favor more lipophilic agents.

The effect of the TP53 status on a cells’ susceptibility to styrylquinolines seems to be especially interesting. Those that were halogenated in the phenyl ring (**5b**, **6b**, **11b** and **12b**) were roughly 2–3 times more effective against HCT116 (p53^-/-^) than against the wild type cells. On the other hand, the acetoxy group in the phenyl ring decreased the activity against the mutants (**2b**-**4b**). Compounds from group **C** had approximately the same level of activity regardless of the p53 status. Only the 2-ethoxy derivative (**1c**) was more active against the p53 null cells in this group, but the differences were too small to draw more serious remarks. On the other hand, the p53 reactivator CP-31398 appeared to be significantly less active against the mutant cells which is in agreement with previous results [24,26].

**Fig 5:**
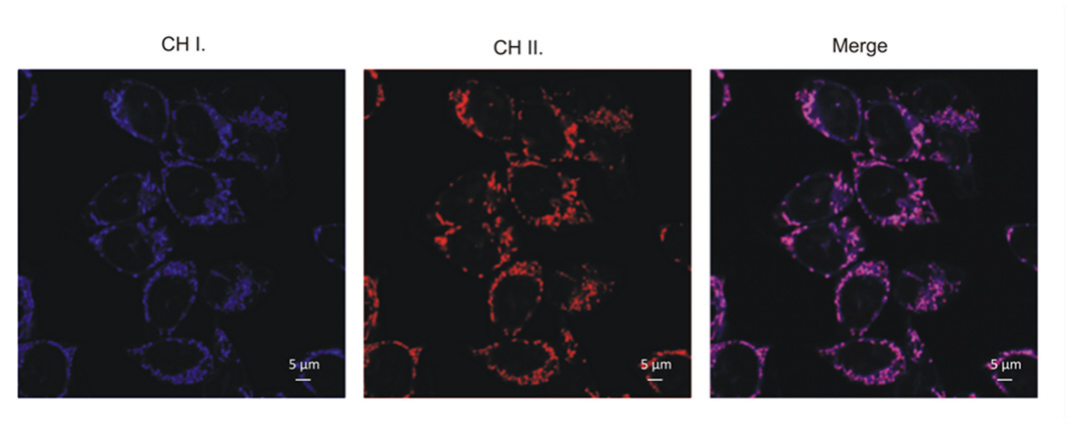
*In vitro* staining of HCT116 cells treated with 6b (CH I.) and MitoTracker Orange CMTMRos dye (CH II.).

A possible explanation is that styrylquinolines can activate a downstream signal cascade in a p53-independent manner. This hypothesis is somewhat supported by lipophilicity—an activity relationship that prefers the more lipophilic compounds (see Fig 4). Slightly more lipophilic and basic compounds may easily penetrate and disorganize the mitochondrial membrane [37] thus affecting the release of the signal transducers that are involved in the intrinsic apoptosis pathway [38]. Fortunately, several of the compounds that were tested showed good fluorescence in the violet-blue wavelengths (see S1 Table). This prompted us to perform cellular localization experiments (Fig 5 and S1 Fig).

The micrographs that are presented show the subcellular localization of the **6b** compound following staining with fluorescent organelle-specific dyes that accumulated selectively in mitochondria. After a two-hour incubation, the compound penetrated into the cell interior efficiently and an intense blue fluorescence that originated from the internalized compound was observed. This is the way that the MitoTracker Orange CMTMRos dye and styrylquinoline accumulates in the mitochondria of healthy cells. Observation of the combined channels confirmed the mitochondrial localization of the studied compounds. If this hypothesis is correct, the post-mitochondrial pathway of apoptosis should be activated.

**Fig 6:**
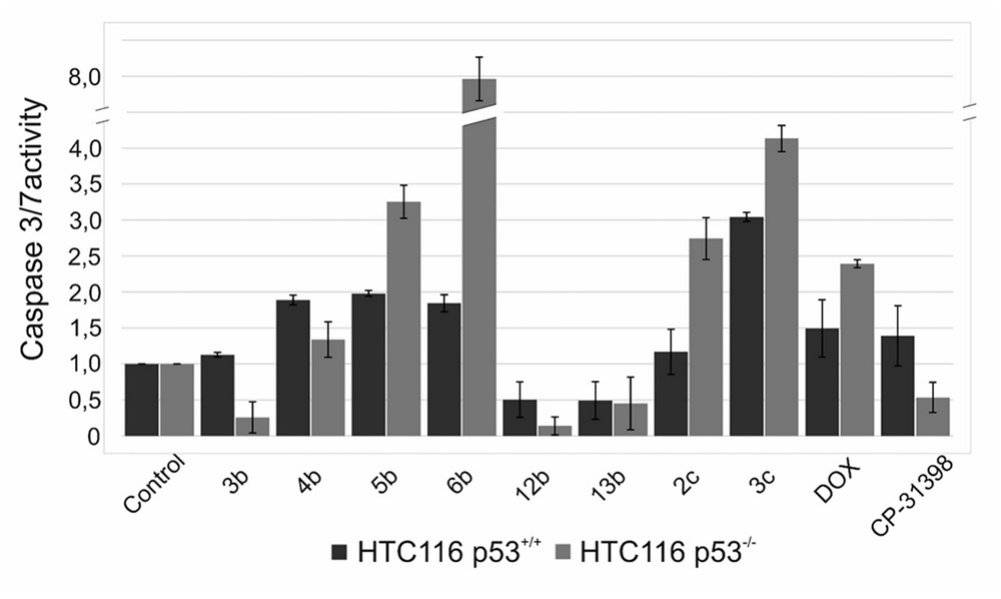
Caspase-3/7 activities on the HCT116 cell lines.

The release of cytochrome c from mitochondria leads to the activation of caspase 9, and subsequently caspases 3 and 7. On the other hand, caspase 9 may be activated by caspase 3 later in an autostimulant feedback [39]. Caspase 3 is responsible for the amplification of the whole cascade, thus its activation is called the “point of no return” [40]. Alternatively, other factors may be released from the mitochondria membrane, e.g., the apoptosis-inducing factor AIF or endonuclease G, which are effectors of a caspase-independent apoptosis that is driven by DNA fragmentation without the activation of the caspase cascade [41]. Wischhusen et al. suggested that CP-31398 exerts some effect on the p53 null cell but “an unusual type of apoptosis in that caspases appeared to play no role” [24]. In order to distinguish between those pathways, we assayed caspase 3/7 activity. Our results confirmed the observations made by Wischhusen as CP-31398 did not increase the activity of caspases in p53^-/-^ cells although it was two times higher than doxorubicin in the wild type cells (Fig 6). On the other hand, styrylquinolines appeared to effectively increase the activity of caspases in a neat correlation to their cytotoxic activity. Apparently, the activity levels for the most active compounds correspond with their ability to induce a higher level of caspases in respective cell lines (compare **6b** and **3c** in Fig 6).

**Fig 7:**
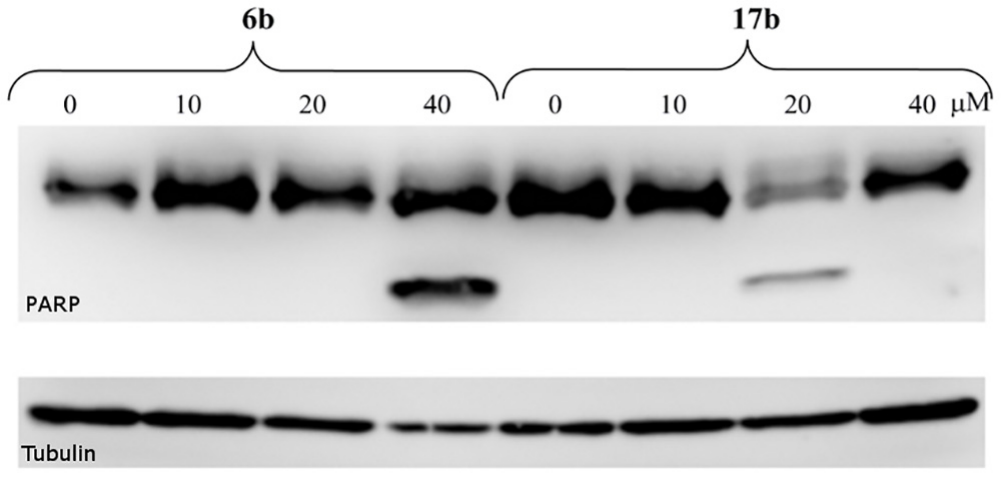
Tubulin level (a) and PARP cleavage (b) in HCT-116 cells for 6b (0, 10, 20, 40 μM) and 17b (0, 10, 20, 40, μM).

During the apoptosis, the natural target for caspases is Polys (ADP-rybose) polymerase-1 PARP-1. This protein is responsible for the detection and repair of DNA damage. It is degraded by caspases during programmed cell death. Cleavage was clearly observable for compounds **6b** and **17b** the PARP as shown in Fig 7.

**Fig 8:**
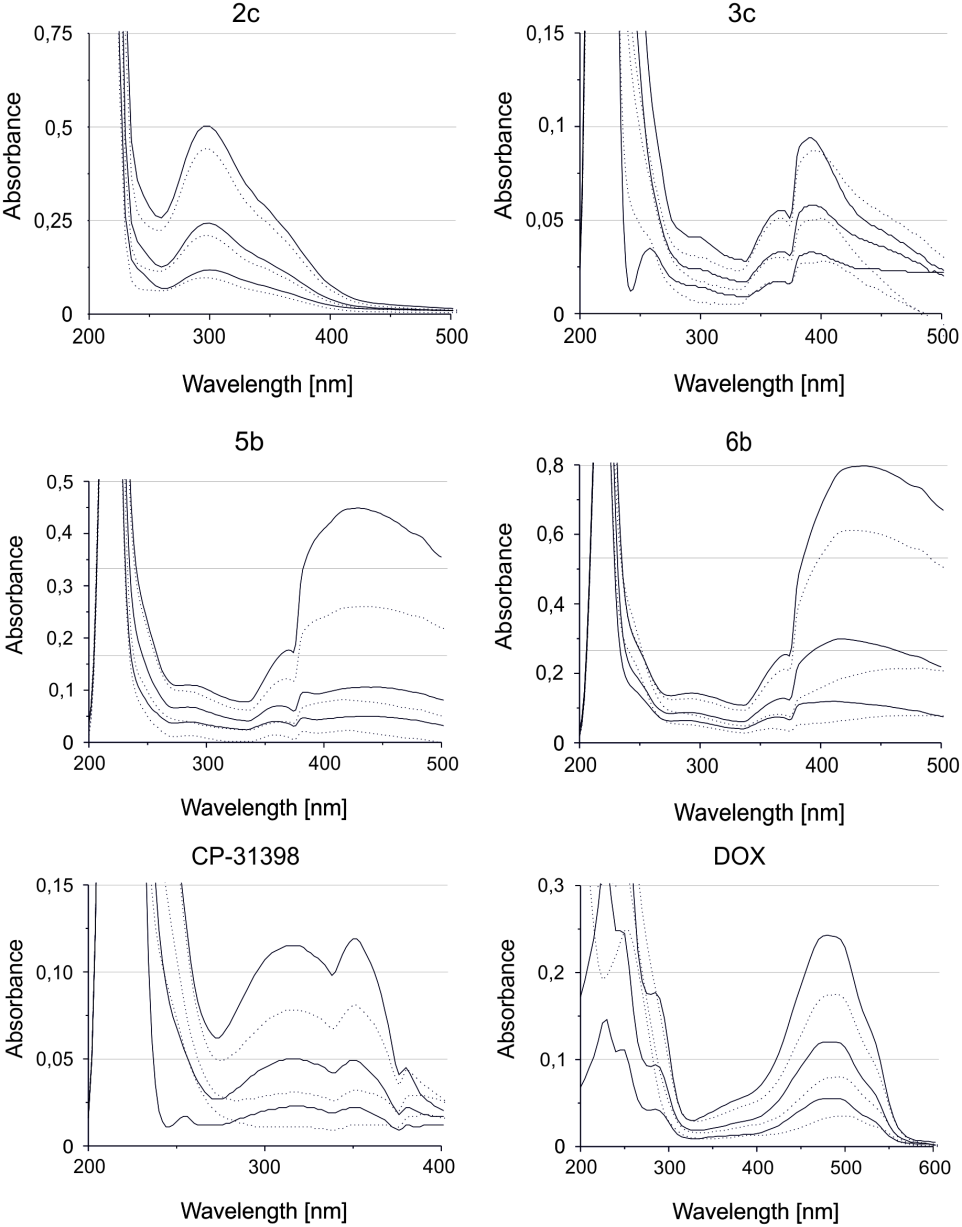
Absorption spectra of 2c, 3c, 5b, 6b, CP-31398 and doxorubicin without CT-DNA (solid line) and with CT-DNA (dotted line).

Large aromatic molecules such as styrylquinolines that have a flat structure are believed to be good DNA intercalators. Based on our results, styrylquinolines may exert their anticancer activity through mitochondrial mtDNA cleavage, similar to doxorubicin, which has been proven to intercalate DNA in the nucleus and mitochondria [42,43]. In order to evaluate this hypothesis, we performed spectrophotometric tests on CT-DNA for the selection of compounds that were tested with referential doxorubicin and CP-31398. The results are shown in Fig 8 and in more detail in S3 Table. As was expected the strongest hypochromism (decrease of intensity spectra) can be observed for **5b** (41.3%) and **6b** (31.0%). Additionally, we also observed a blue shift of 14 nm in the wavelength, which is characteristic for the DNA-bond of these compounds. Similar to **doxorubicin** and **CP-31398**, we observed a strong decrease in the absorption intensity 34.2% and 41.4%, 37.4%, respectively. Furthermore, the interaction of **doxorubicin** with DNA was accompanied by a shift towards higher wavelengths– 10 nm (red shift).

These results may also elucidate a higher activity of **5b** and **6b** against HCT116 mutant cells. Under normal conditions, p53 is responsible for cell cycle arrest and DNA repair when necessary. A lack of this protein may increase the vulnerability of cells to DNA damage. As was recently reported, the p53 null cells undergo apoptosis after treatment with DNA damaging agents while the wild type cells survive after G2 arrest and subsequent DNA repair [44]. With this in mind, strongly intercalating agents such as **5b** and **6b** can affect the mutant cells with stronger effect.

## Conclusions

To conclude, a series of twenty-six styrylquinolines was obtained and tested for their antiproliferative activity. Several of the compounds that were tested showed activity at a micromolar level that was comparable or better than doxorubicin. A positive dependence between lipophilicity and activity was observed. Despite the structural similarity to the known p53 activator CP-31398, the styrylquinolines appeared to be active against cancer cells regardless of their p53 status. The observation of subcellular accumulations and caspase activation may suggest a mechanism of action that is independent of p53 and based on mitochondrial disintegration. This was confirmed by intercalation experiments with CT-DNA. Compounds **5b** and **6b** appeared to be especially interesting leading structures for developing novel anticancer agents against drug-resistant lines.

## Supporting Information

S1 Fig Live imaging of HCT116 (p53^+/+^) cells following 2h incubation with 6b (CH I.). LysoTracker Yellow-HCK-123 and MitoTracker Orange CMTMRos were used for organelle staining (CH II.). Scale bar = 50 μm, and Table DNA binding properties of styrylquinolines
(PDF)

S1 File The chemical characterization of the obtained compounds, the exemplary NMR spectra and physicochemical structure information. (PDF)

S1 Table Fluorescent and absorbtion properties of the tested compounds in DMSO. (PDF)

S2 Table Comparison of cytotoxicity measured by means MTS and trypan blue methods (PDF)

S3 Table DNA binding properties of tested styrylquinolines. (PDF)
