# Supplementary material for: Design, Synthesis and In Vitro Activity of Anticancer Styrylquinolines. The p53 Independent Mechanism of Action
Source: PLoS One. 2015 Nov 23;10(11):e0142678. doi: 10.1371/journal.pone.0142678 (PMC4657899; doi:10.1371/journal.pone.0142678)
Supplement: S1 Fig — LysoTracker Yellow-HCK-123 and MitoTracker Orange CMTMRos were used for organelle staining (CH II.). Scale bar = 50 μm, and Table DNA binding properties of styrylquinolines (PDF) [file pone.0142678.s001.pdf]

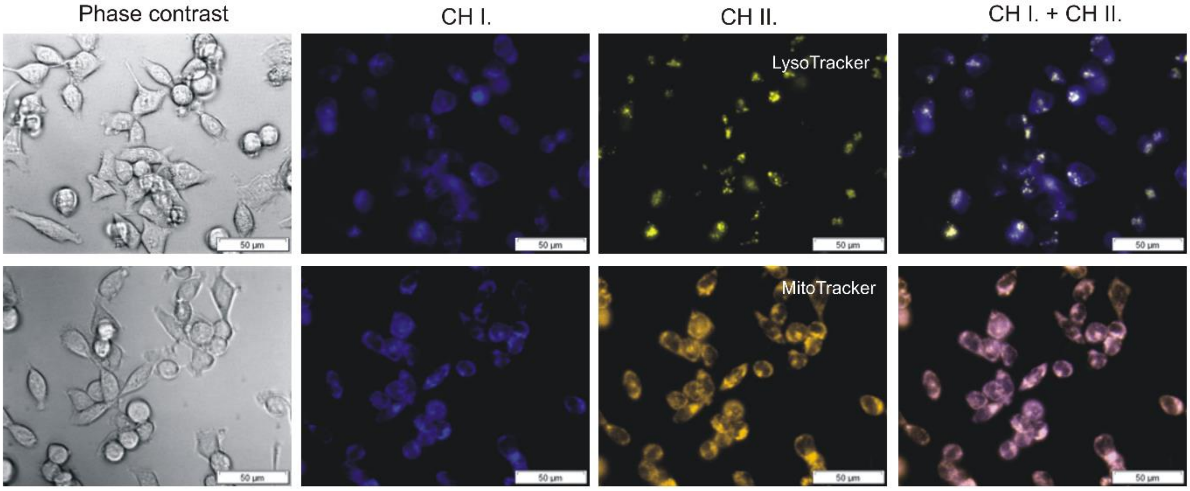

S1 Figure. Live imaging of HCT116 (p53$^{+/+}$) cells following 2h incubation with **6b** (CH I.). LysoTracker Yellow-HCK-123 and MitoTracker Orange CMTMRos were used for organelle staining (CH II.). Scale bar = 50 µm.
