# Supplementary material for: Design, Synthesis and In Vitro Activity of Anticancer Styrylquinolines. The p53 Independent Mechanism of Action
Source: PLoS One. 2015 Nov 23;10(11):e0142678. doi: 10.1371/journal.pone.0142678 (PMC4657899; doi:10.1371/journal.pone.0142678)
Supplement: S1 File — (PDF) [file pone.0142678.s002.pdf]

S1 Chemistry

S1.1 NMR data, yields, HRMS and melting points of all products2 – [2-(2-Hydroxyphenyl)vinyl]quinoline(**1a**). Yield 6.1% of a crystalline compound, mp 205ºC [1].

2 – [2-(4-Ethoxyphenyl)vinyl]quinoline(**2a**). Yield 17% of a yellow solid, mp 143-145ºC [1].

2 – [2-(4-Buthoxyphenyl)vinyl]quinoline(**3a**). Method A, a beige solid, yield 24%, 93ºC, ^{1}H NMR (400 MHz, $CDCl_3$) δ 8.13 (d, *J* = 8.6 Hz, 1H, Ar-H), 8.09 (d, *J* = 8.6 Hz, 1H, Ar-H), 7.80 (d, *J* = 8.0 Hz, 1H, Ar-H), 7.73 (d, *J* = 8.3 Hz, 1H, Ar-H), 7.71 – 7.67 (m, 1H, Ar-H), 7.65 (d, *J* = 7.2 Hz, 1H, Ar-H), 7.60 (d, *J* = 8.7 Hz, 2H, Ar-H), 7.50 (t, *J* = 7.5 Hz, 1H, Ar-H), 7.33 (s, 1H, Ar-H), 6.95 (d, *J* = 8.7 Hz, 2H, Ar-H), 4.03 (t, *J* = 6.5 Hz, 2H, OCH_2), 1.86 – 1.77 (m, 2H, CH_2), 1.59 – 1.48 (m, 2H, CH_2), 1.01 (t, *J* = 7.4 Hz, 3H, CH_3); ^{13}C NMR (101 MHz, $CDCl_3$) δ 159.09, 157.41, 135.51, 134.74, 130.62, 129.59, 128.89, 128.71, 127.51, 127.47, 127.14, 126.89, 126.40, 122.25, 118.97, 114.86, 114.24, 67.76, 31.30, 19.24, 13.84; m/z = 304.34 $[M+H]^+$; Anal. calcd. for $C_{21}H_{21}NO$: C, 83.13; H, 6.98; N, 4.62; O, 5.27; found: C, 82.79; H, 6.65; N, 4.79; O, 5.06.

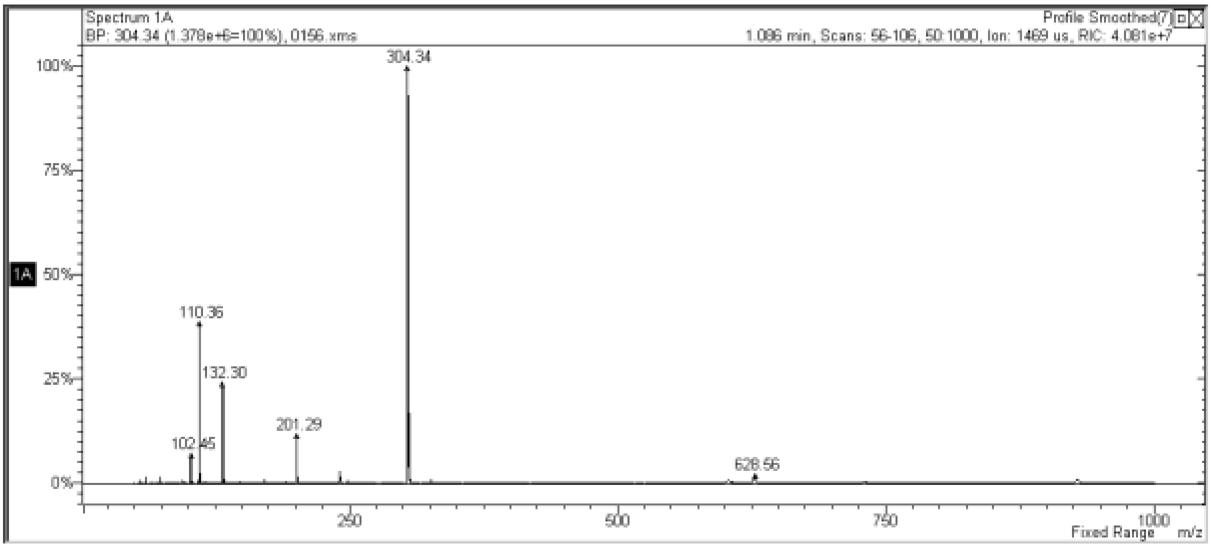

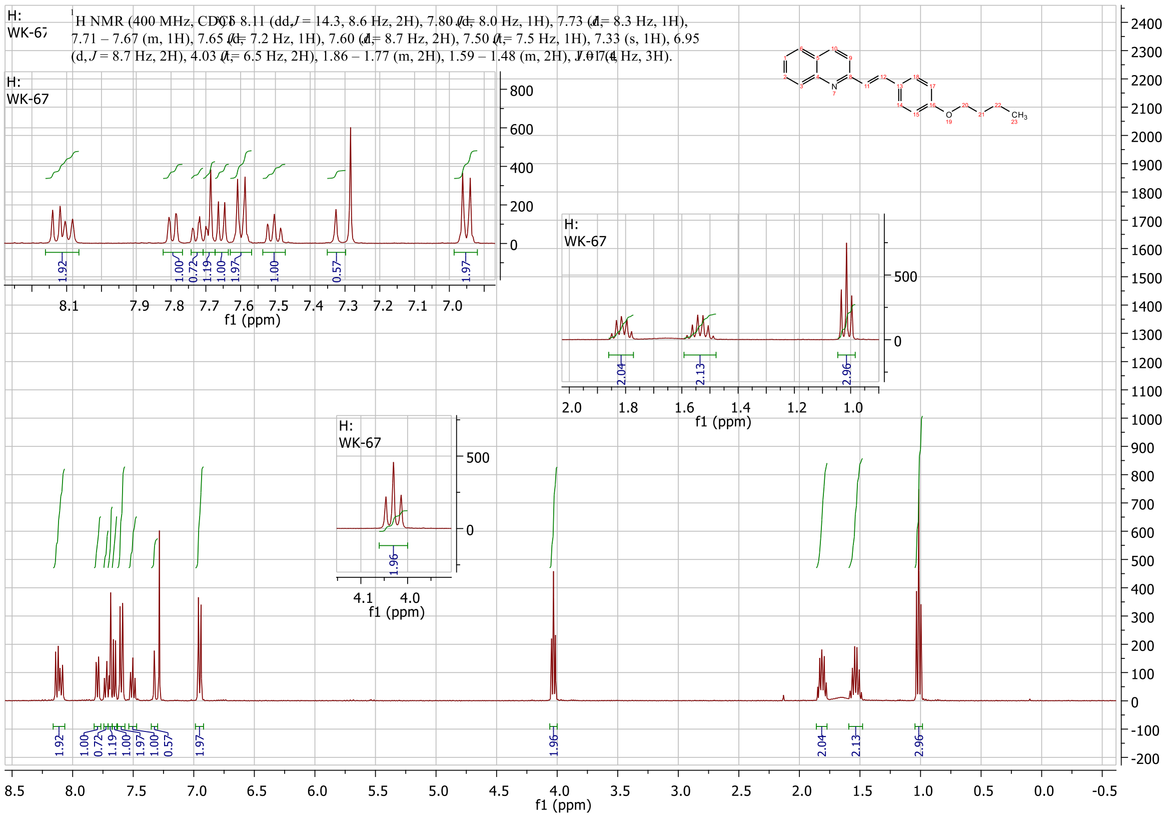

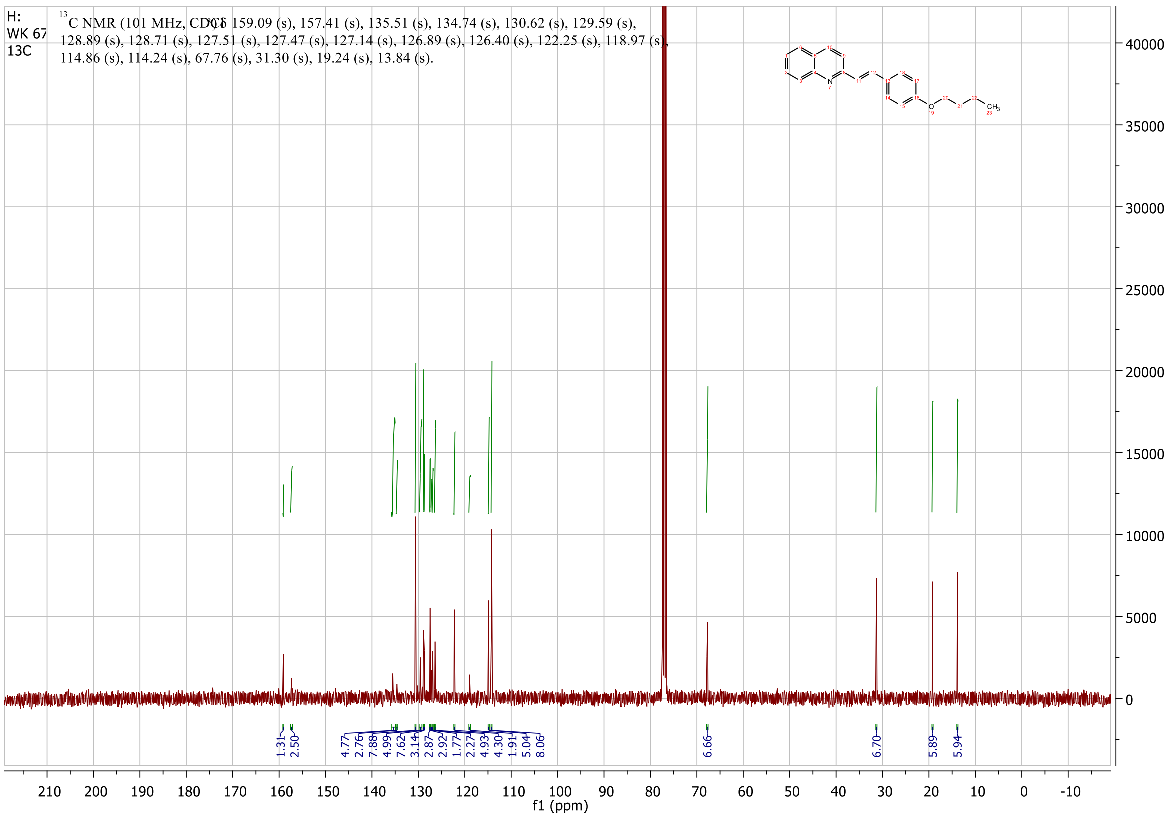

2 – [2-(3,5-Dimethoxyphenyl)vinyl]quinolin-8-ol (1b). Yield 57% of a beige crystals; mp 122°C [2].

2 – (2-(8-Acetoxyquinolin-2-yl)vinyl)phenyl acetate (2b). Method A, a beige solid, yield 94%, mp 128°C, ^{1}H NMR (400 MHz, DMSO) δ 8.43 (d, *J* = 8.6 Hz, 1H, Ar-H), 7.97 (dd, *J* = 7.7, 1.4 Hz, 1H, Ar-H), 7.91 – 7.85 (m, 2H, Ar-H), 7.78 (d, *J* = 16.3 Hz, 1H, C=C-H), 7.59 (d, *J* = 7.6 Hz, 1H, Ar-H), 7.57 – 7.52 (m, 1H, Ar-H), 7.50 (d, *J* = 16.2 Hz, 1H, C=C-H), 7.44 (td, *J* = 7.8, 1.6 Hz, 1H, Ar-H), 7.37 (t, *J* = 7.1 Hz, 1H, Ar-H), 7.22 (dd, *J* = 8.0, 1.2 Hz, 1H, Ar-H), 2.48 (s, 3H, CH_3), 2.42 (s, 3H, CH_3); ^{13}C NMR (101 MHz, DMSO) δ 169.70, 155.37, 149.14, 147.56, 140.78, 137.34, 131.04, 130.26, 129.19, 128.83, 127.86, 127.47, 126.87, 126.57, 126.15, 123.76, 122.18, 121.85, 21.21, 21.10; m/z = 349.56 $[M+2H]^+$; Anal. calcd. for $C_{21}H_{17}NO_4$: C, 72.61; H, 4.93; N, 4.03; O, 18.42; found: C, 72.78; H, 4.57; N, 4.01; O, 18.81.

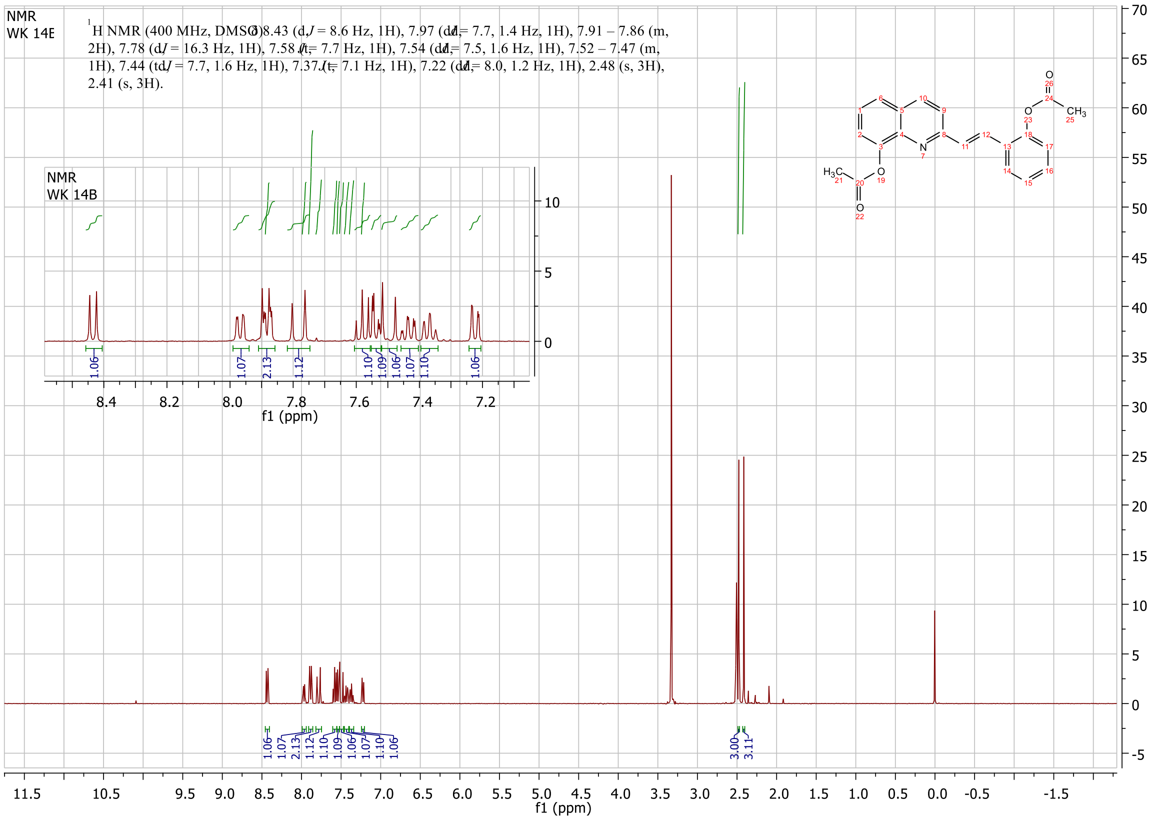

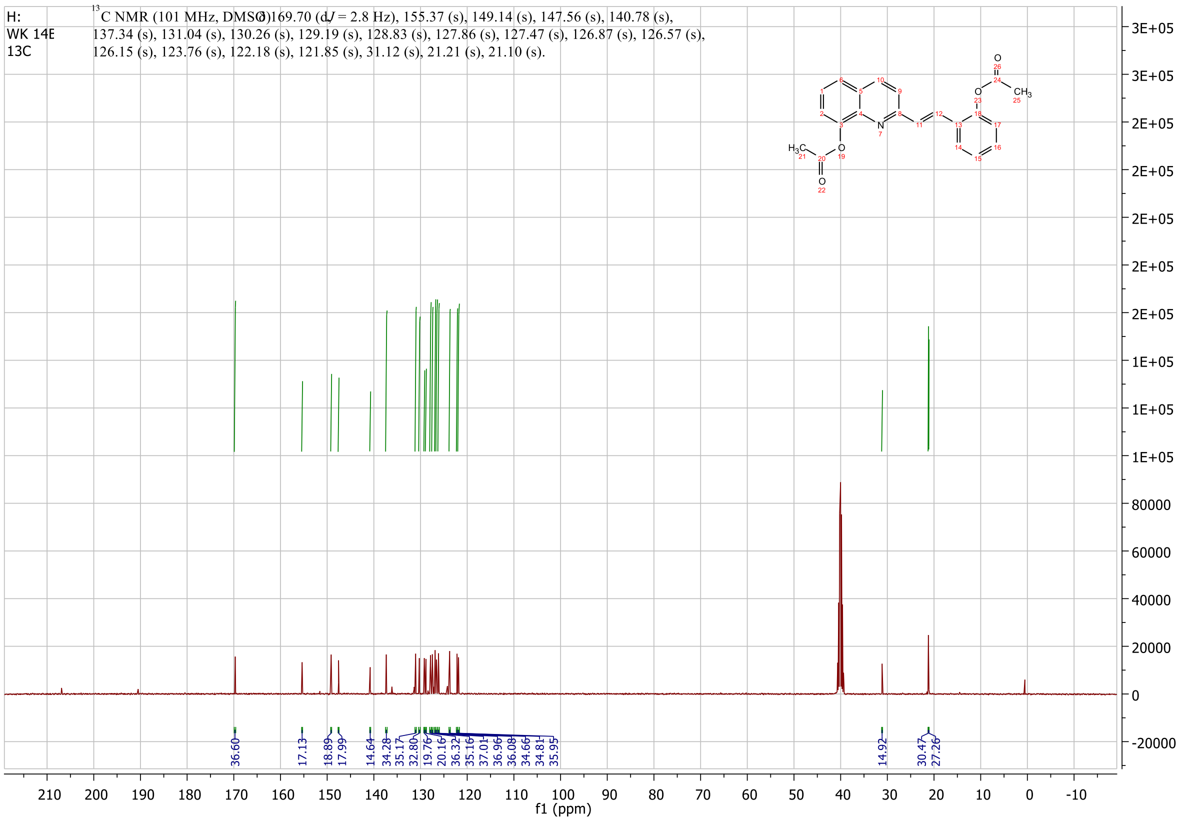

2 – [2-(2-Acetoxyphenyl)vinyl]quinolin-8-ol (3b). Method B, white crystals, yield 28%, mp 128°C, ^{1}H NMR (400 MHz, DMSO) δ 9.65 (bs, 1H, OH), 8.31 (d, *J* = 8.6 Hz, 1H, Ar-H), 7.99 (d, *J* = 16.3 Hz, 1H, C=C-H), 7.92 (dd, *J* = 7.7, 1.6 Hz, 1H, Ar-H), 7.84 (d, *J* = 8.6 Hz, 1H, Ar-H), 7.50 (d, *J* = 16.3 Hz, 1H, C=C-H), 7.45 – 7.33 (m, 4H, Ar-H), 7.21 (dd, *J* = 7.9, 1.3 Hz, 1H, Ar-H), 7.10 (dd, *J* = 7.1, 1.8 Hz, 1H, Ar-H), 2.44 (s, 3H, CH_3); ^{13}C NMR (101 MHz, DMSO) δ 169.94, 153.59, 153.52, 149.10, 138.70, 137.05 , 130.94, 129.94, 129.54, 128.35, 127.90, 127.76, 127.51, 126.81, 123.86, 121.53, 118.08, 111.93, 21.40; m/z = 306.58 $[M+2H]^+$;Anal. calcd. for $C_{19}H_{15}NO_3$: C, 74.74; H, 4.95; N, 4.59; O, 15.72; found: C, 74.35; H, 4.55; N, 4.59; O, 16.13.

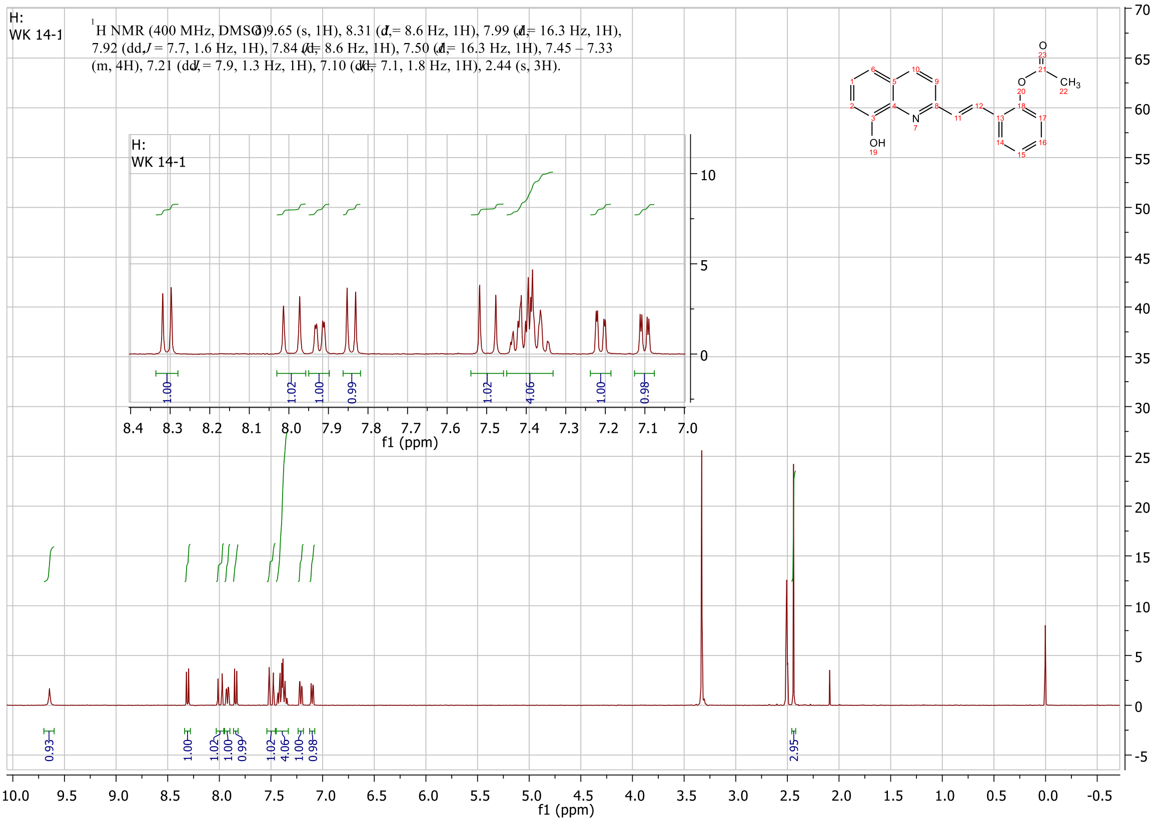
H:
WK 14-1
1H NMR (400 MHz, DMSO) δ 9.65 (s, 1H), 8.31 (d, J = 8.6 Hz, 1H), 7.99 (d, J = 16.3 Hz, 1H), 7.92 (dd, J = 7.7, 1.6 Hz, 1H), 7.84 (d, J = 8.6 Hz, 1H), 7.50 (d, J = 16.3 Hz, 1H), 7.45 – 7.33 (m, 4H), 7.21 (dd, J = 7.9, 1.3 Hz, 1H), 7.10 (dd, J = 7.1, 1.8 Hz, 1H), 2.44 (s, 3H).
f1 (ppm)

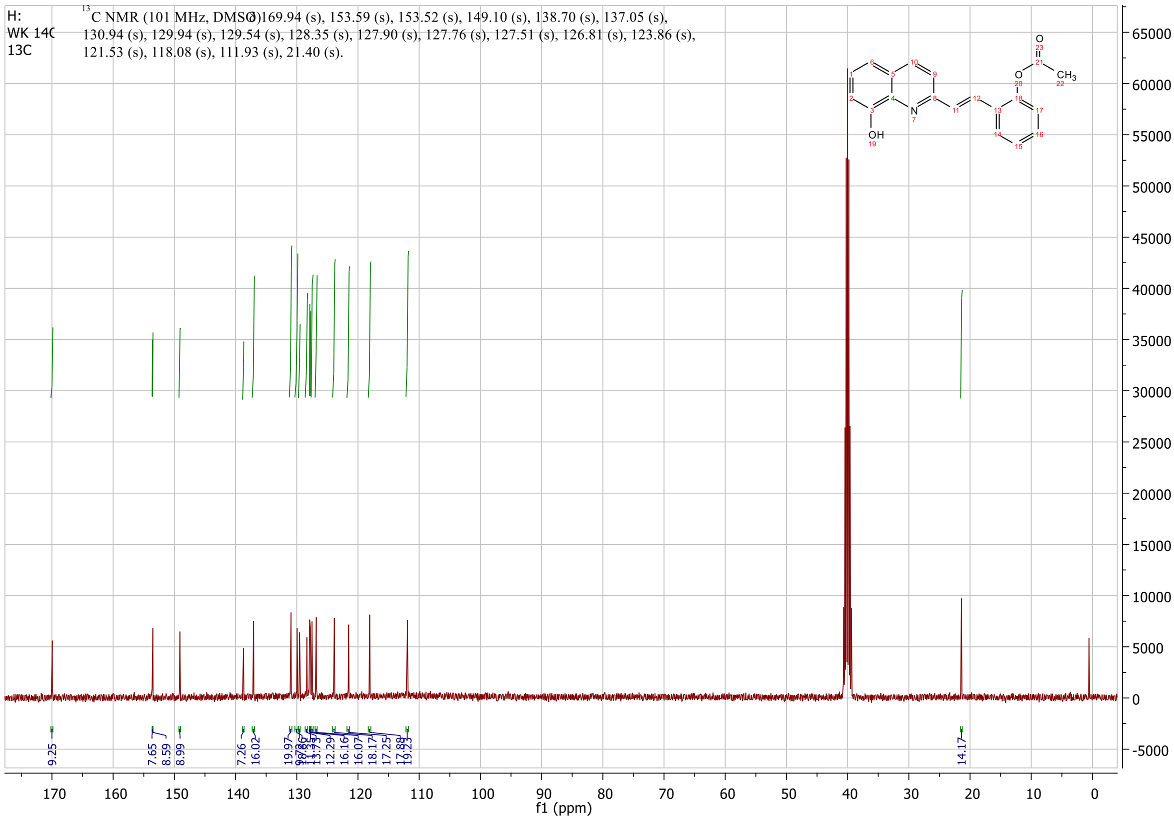
H:
WK 14C
13C
13C NMR (101 MHz, DMSO) δ 169.94 (s), 153.59 (s), 153.52 (s), 149.10 (s), 138.70 (s), 137.05 (s), 130.94 (s), 129.94 (s), 129.54 (s), 128.35 (s), 127.90 (s), 127.76 (s), 127.51 (s), 126.81 (s), 123.86 (s), 121.53 (s), 118.08 (s), 111.93 (s), 21.40 (s).
f1 (ppm)

2 – [2-(3-Acetoxyphenyl)vinyl]quinolin-8-ol (4b). Method B, a yellow solid, yield 29%, mp 83°C, ^{1}H NMR (400 MHz, DMSO) δ 9.57 (bs, 1H, OH), 8.31 (d, *J* = 8.6 Hz, 1H, Ar-H), 8.15 (d, *J* = 16.2 Hz, 1H, C=C-H), 7.77 (d, *J* = 8.5 Hz, 1H, Ar-H), 7.60 (d, *J* = 7.8 Hz, 1H, Ar-H), 7.50 (dd, *J* = 14.2, 9.9 Hz, 3H, Ar-H), 7.44 – 7.35 (m, 2H, Ar-H), 7.16 – 7.07 (m, 2H, Ar-H), 2.32 (s, 3H, CH_3); ^{13}C NMR (101 MHz, DMSO) δ 169.70, 153.55, 153.44, 151.48, 138.61, 137.06, 133.79, 130.44, 129.43, 128.24, 127.67, 125.24, 122.35, 121.61, 120.53, 118.04, 111.69, 21.36; m/z = 307.37 $[M+2H]^+$; Anal. calcd. for $C_{19}H_{15}NO_3$: C, 74.74; H, 4.95; N, 4.59; O, 15.72; found: C, 74.88; H, 4.57; N, 4.66; O, 15.91.

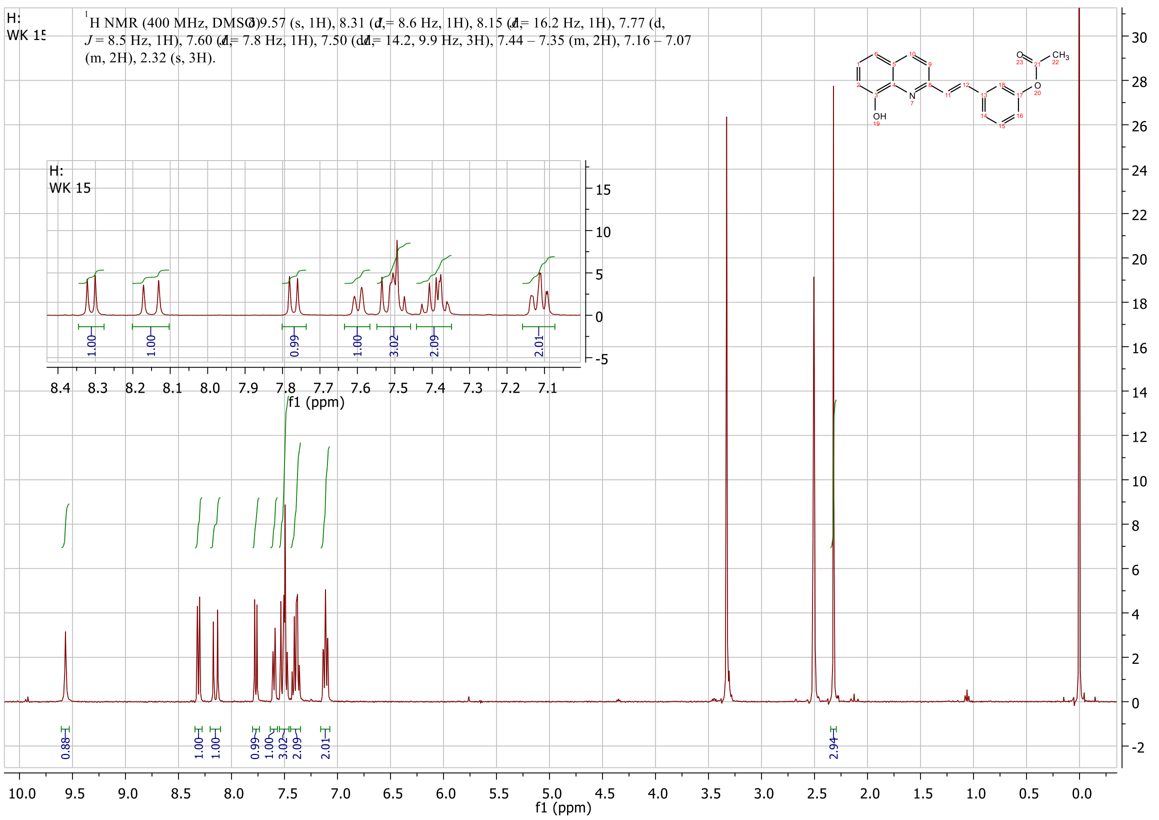

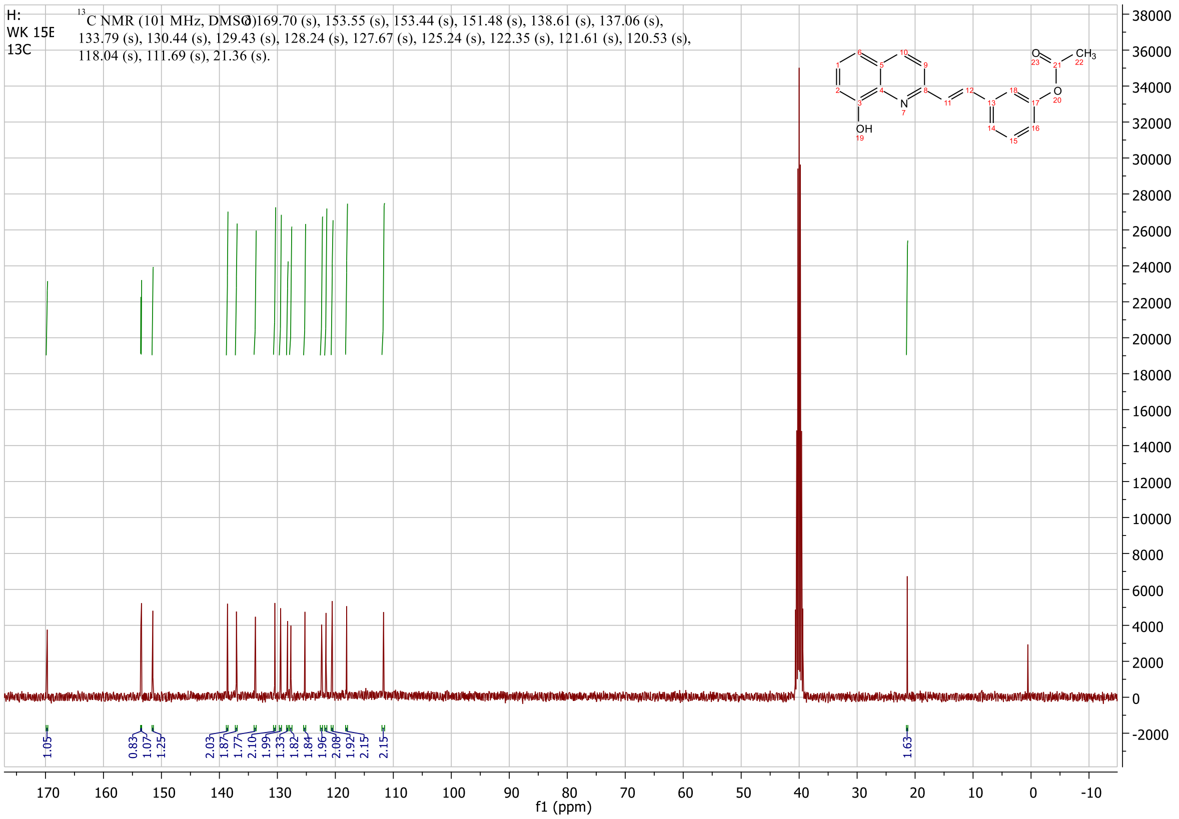

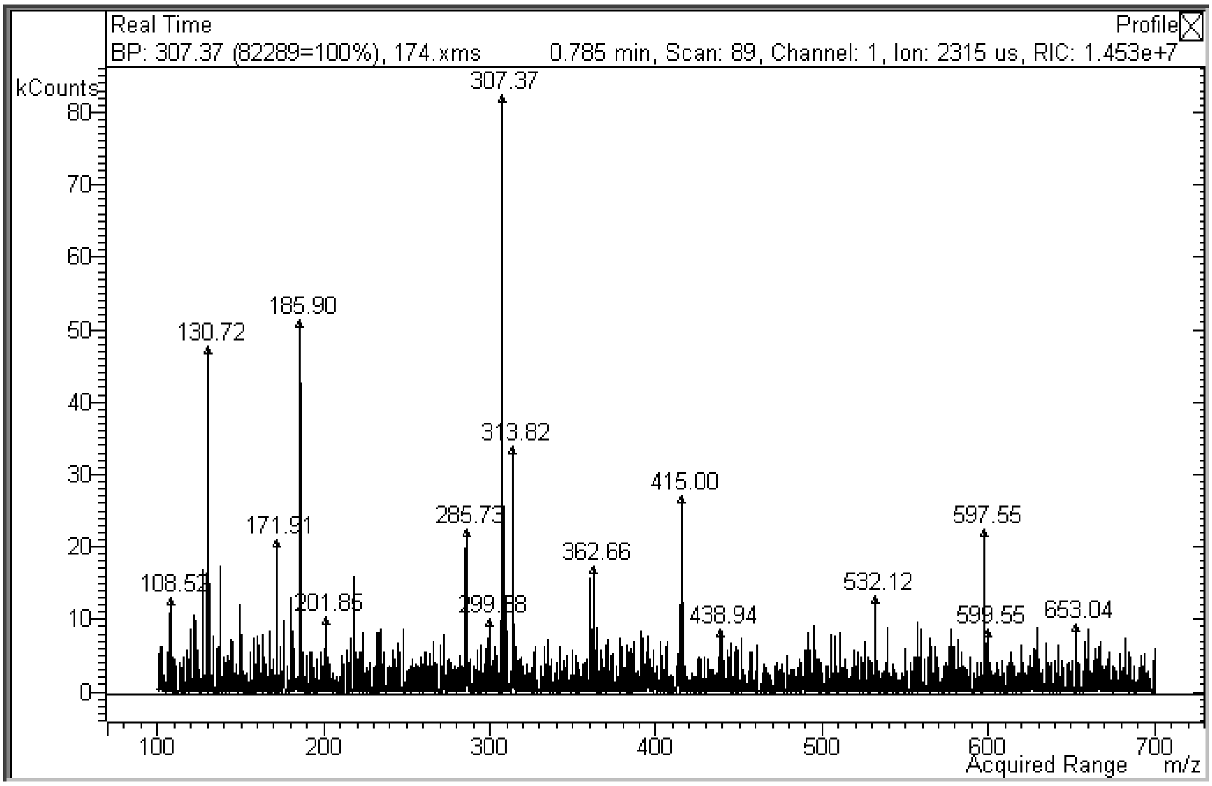

2 – [2-(2,3-Dichlorophenyl)vinyl]quinolin-8-ol (**5b**).Yield 94% of beige crystals; mp 148°C [2].

2 – [2-(3,4-Dichlorophenyl)vinyl]quinolin-8-ol (**6b**).Yield 96.9% of yellow crystals, mp 140°C [3].

2 – [2-(3,4-Diacetoxy-5-methoxyphenyl)vinyl]quinolin-8-ol (7b). Method A, a yellow solid, yield 72%, mp 172°C, ^{1}H NMR (400 MHz, DMSO) δ 8.44 (d, *J* = 8.7 Hz, 1H, Ar-H), 7.87 (dd, *J* = 10.6, 4.9 Hz, 2H, Ar-H), 7.79 (d, *J* = 16.2 Hz, 1H, C=C-H), 7.58 (t, *J* = 7.7 Hz, 1H, Ar-H), 7.51 (ddd, *J* = 18.0, 5.2, 1.5 Hz, 2H, Ar-H), 7.44 (d, *J* = 1.5 Hz, 1H, Ar-H), 7.27 (d, *J* = 1.5 Hz, 1H, Ar-H), 3.91 (s, 3H, OCH_3), 2.32 (s, 3H, CH_3), 2.30 (s, 3H, CH_3); ^{13}C NMR (101 MHz, DMSO) δ 169.88, 168.69, 168.05, 155.57, 152.79 , 147.53, 143.85, 140.88, 137.29, 135.14, 133.85, 132.29, 130.23, 128.74, 126.34, 122.31, 121.22, 114.79, 109.28, 56.87, 21.23, 20.81; m/z = 395.70 $[M+2H]^{+}$; Anal. calcd. for $C_{22}H_{19}NO_6$: C, 67.17; H, 4.87; N, 3.56; O, 24.40; found: C, 67.02; H, 4.52; N, 3.24; O, 24.79.

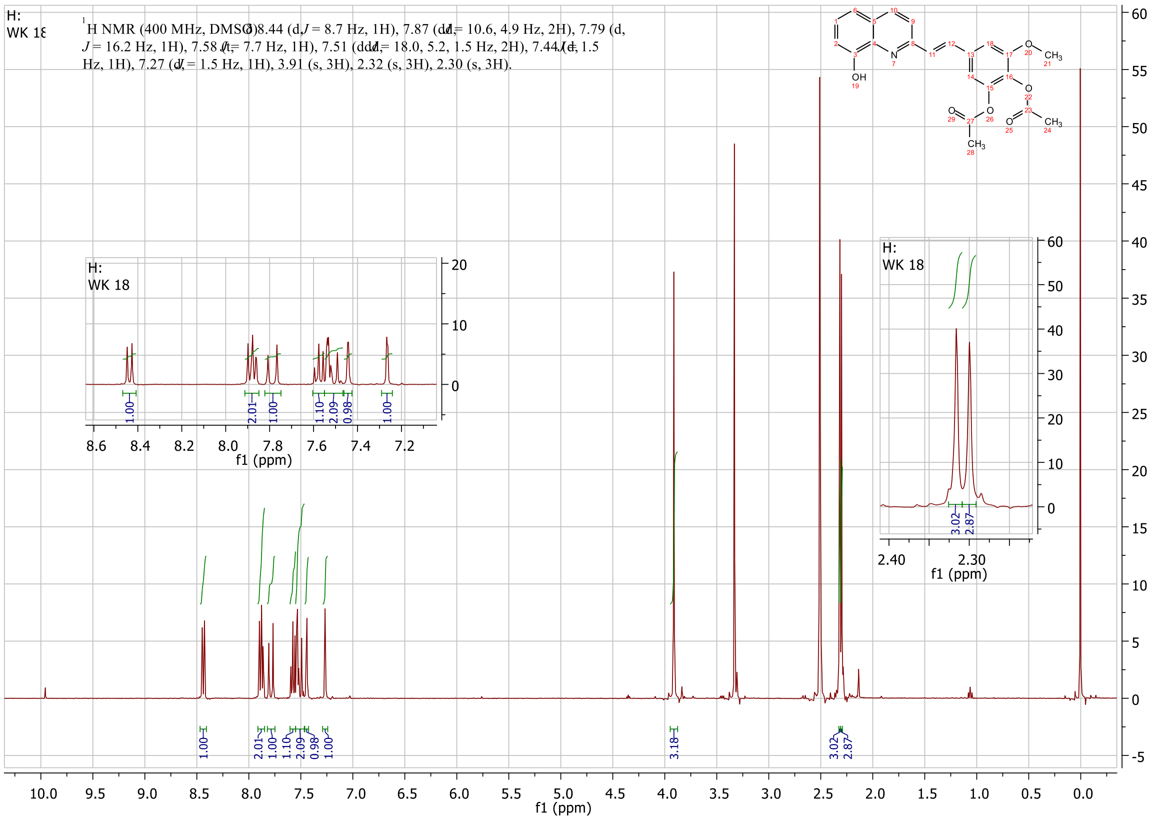

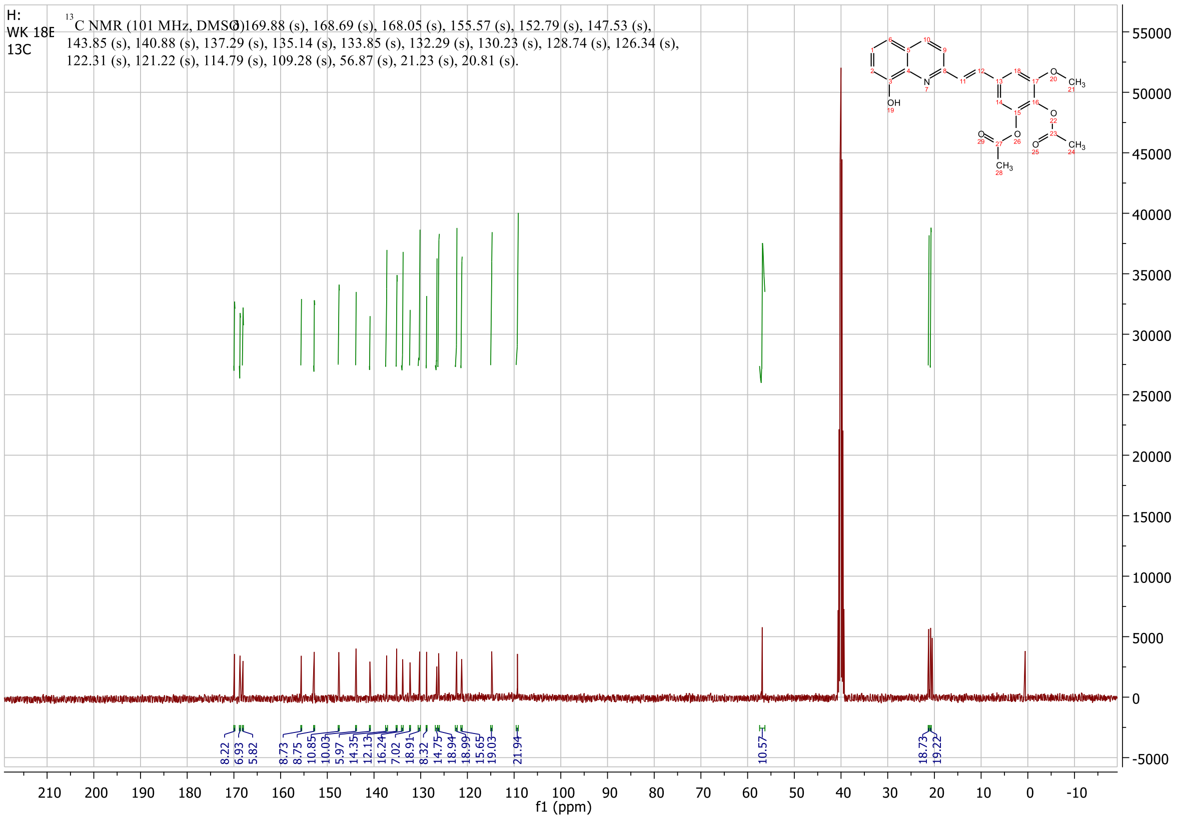

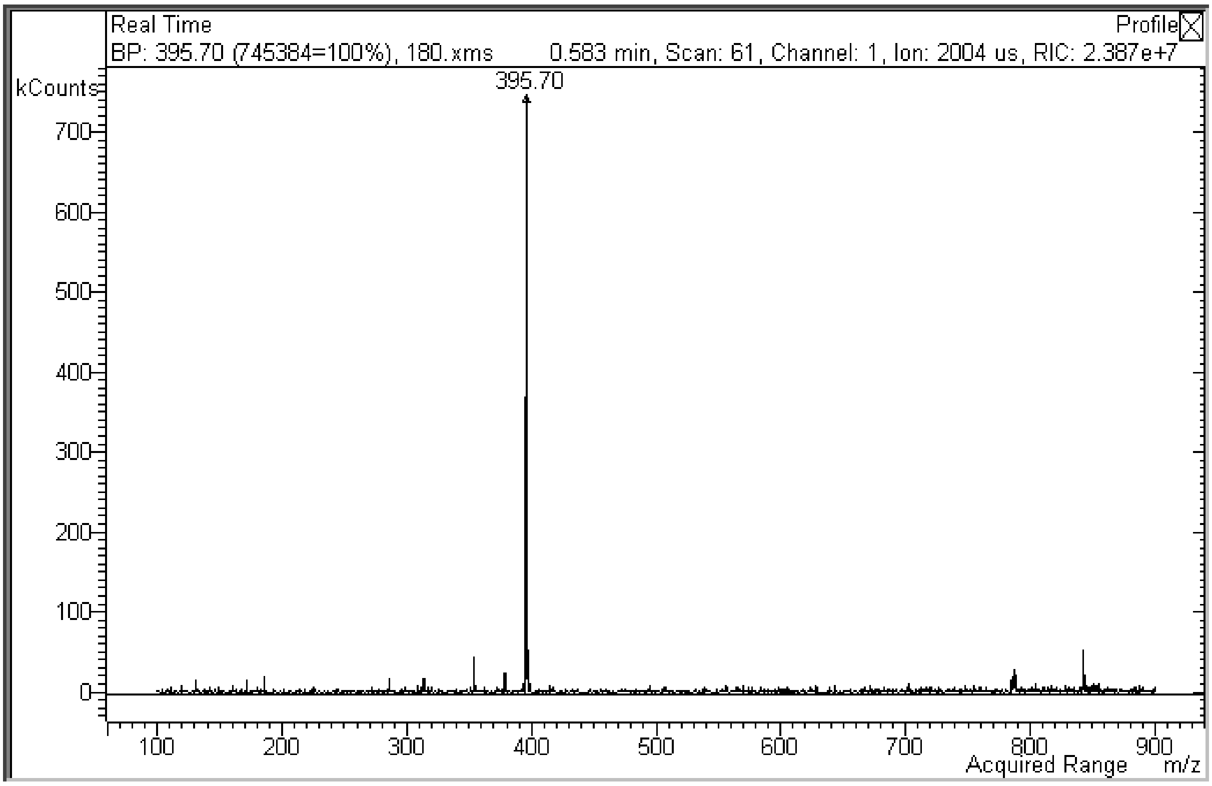

4 – (2-(8-Acetoxyquinolin-2-yl)vinyl)-1,3-phenylene diacetate (8b). Method A, a yellow solid, yield 81%, mp 159°C, ^{1}H NMR (400 MHz, DMSO) δ 8.43 (d, J = 8.6 Hz, 1H, Ar-H),

8.01 (d, J = 8.6 Hz, 1H, Ar-H), 7.91 – 7.85 (m, 2H, Ar-H), 7.75 (d, J = 16.3 Hz, 1H, C=C-H), 7.61 – 7.52 (m, 2H, Ar-H), 7.48 (d, J = 16.2 Hz, 1H, C=C-H), 7.17 (dd, J = 8.5, 2.4 Hz, 1H, Ar-H), 7.13 (d, J = 2.3 Hz, 1H, Ar-H), 2.47 (s, 3H, CH_3), 2.41 (s, 3H, CH_3), 2.30 (s, 3H, CH_3); ^{13}C NMR (101 MHz, DMSO) δ 169.70, 169.50, 169.36, 155.32, 151.40, 149.32, 147.54, 140.76, 137.36, 131.04, 128.83, 128.07, 127.14, 126.91, 126.60, 126.16, 122.19, 121.83, 120.61, 117.51, 21.31, 21.20, 21.10; m/z = 407.75 [M+2H]$^+$; Anal. calcd. for $C_{23}H_{19}NO_6$: C, 68.14; H, 4.72; N, 3.46; O, 23.68; found: C, 68.17; H, 4.55; N, 3.49; O, 23,80.

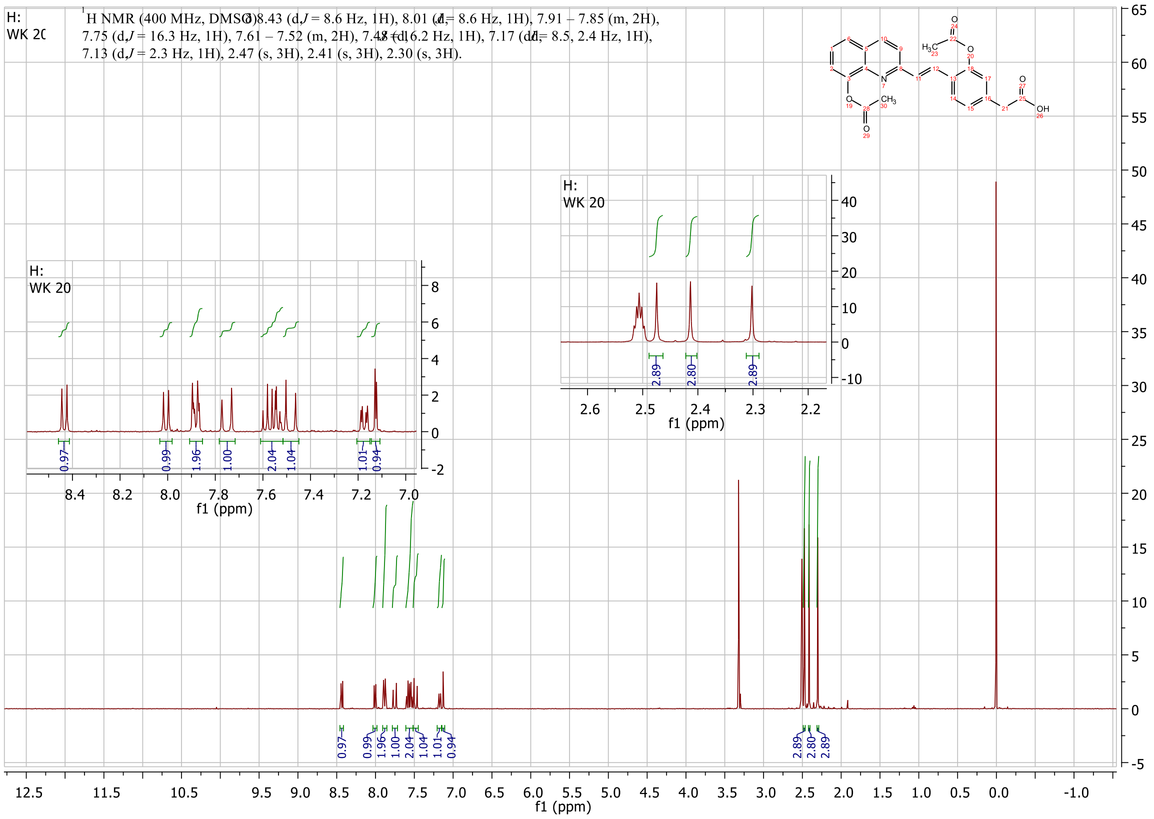

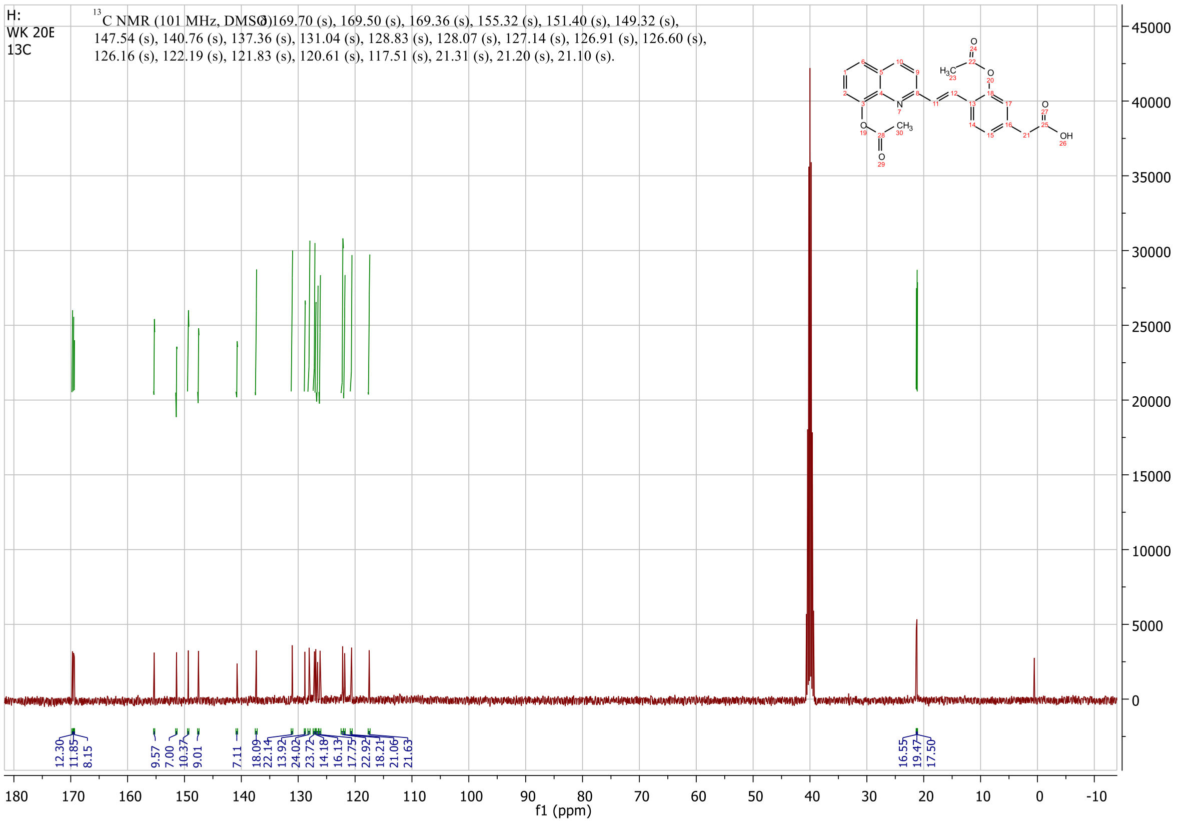

2 – [2-(2,4,6-Trihydroxyphenyl)vinyl]quinolin-8-ol (**9b**). Method C, a brown solid, yield 93%, mp 279°C, ^{1}H NMR (400 MHz, DMSO) δ 11.92 (bs, 1H, OH), 10.02 (bs, 1H, OH), 9.65 (bs, 1H, OH), 8.79 (d, *J* = 9.1 Hz, 1H, Ar-H), 8.45 (d, *J* = 9.2 Hz, 1H, Ar-H), 8.27 (d, *J* = 16.2 Hz, 1H, C=C-H), 7.96 (d, *J* = 16.3 Hz, 1H, C=C-H), 7.64 – 7.55 (m, 2H, Ar-H), 7.50 – 7.43 (m, 1H, Ar-H), 7.04 (d, *J* = 8.7 Hz, 1H, Ar-H), 6.53 (t, *J* = 10.0 Hz, 1H, Ar-H); ^{13}C NMR (101 MHz, DMSO) δ 154.07, 150.56, 148.35, 148.28, 143.97, 141.88, 133.57, 129.33, 128.51, 127.89 , 120.97, 118.85, 118.80, 116.80, 115.84, 115.37, 109.12; m/z = 318.24 [M+Na]$^{+}$; Anal. calcd. for $C_{17}H_{13}NO_4$: C, 69.15; H, 4.44; N, 4.74; O, 21.67; found: C, 68.78; H, 4,23; N, 4.52; O, 21.90.

2 – (2-(8-Acetoxyquinolin-2-yl)vinyl)benzene-1,3,5-triyl triacetate (10b). Method A, a beige solid, yield 41%, mp 189°C, ^{1}H NMR (400 MHz, DMSO) δ 8.44 (d, *J* = 8.6 Hz, 1H, Ar-H), 7.95 – 7.85 (m, 2H, Ar-H), 7.57 (qd, *J* = 7.5, 3.0 Hz, 3H, Ar-H), 7.38 (d, *J* = 16.5 Hz, 1H, C=C-H), 7.13 (s, 2H, Ar-H), 2.45 (s, 3H, CH_3), 2.37 (s, 6H, CH_3), 2.30 (s, 3H, CH_3); ^{13}C NMR (101 MHz, DMSO) δ 169.60, 169.16, 169.12, 155.05, 150.43, 149.91, 147.58, 140.65, 137.51, 134.23, 128.96, 126.76, 126.17, 123.67, 122.16, 121.84, 120.98, 116.29, 115.65, 31.13, 21.26,

21.18, 21.01; m/z = 465.98 [M+2H]$^+$; Anal. calcd. for $C_{25}H_{21}NO_8$: C, 64.79; H, 4.57; N, 3.02; O, 27.62; found: C, 64.94; H, 4.21; N, 2.99; O, 27.97.

2 – [2-(2-Fluorophenyl)vinyl]quinolin-8-ol (**11b**). Method C, an orange solid, yield 49%, mp 239°C, ^{1}H NMR (400 MHz, DMSO) δ 11.46 (bs, 1H, OH), 8.81 (d, *J* = 7.9 Hz, 1H, Ar-H), 8.41 (d, *J* = 7.1 Hz, 1H, Ar-H), 8.24 (d, *J* = 16.5 Hz, 1H, C=C-H), 8.01 (d, *J* = 16.4 Hz, 1H, C=C-H), 7.83 (t, *J* = 7.7 Hz, 1H, Ar-H), 7.66 – 7.56 (m, 2H, Ar-H), 7.52 (dd, *J* = 12.9, 6.6 Hz, 1H, Ar-H), 7.44 (d, *J* = 5.8 Hz, 1H, Ar-H), 7.38 (dd, *J* = 13.3, 4.9 Hz, 2H, Ar-H); ^{13}C NMR (101 MHz, DMSO) δ 162.39, 159.88, 152.35, 149.87, 143.78, 134.68, 132.80, 130.00, 128.86, 125.71, 124.76, 123.43, 119.69, 118.56, 116.96, 116.74, 116.15; m/z = 266.24 [M+2H]$^+$; Anal. calcd. for $C_{17}H_{12}FNO$:C, 76.97; H, 4.56; F, 7.16; N, 5.28; O, 6.03; found: C, 76.57; H, 4.19; N, 4.95.

2 – (2-Fluorostyryl)quinolin-8-yl acetate (12b). Method A, a yellow solid, yield 78%, mp 117°C, ^{1}H NMR (400 MHz, DMSO) δ 8.44 (d, *J* = 8.6 Hz, 1H, Ar-H), 7.98 (d, *J* = 16.2 Hz, 1H, C=C-H), 7.93 (dd, *J* = 8.0, 1.4 Hz, 1H, Ar-H), 7.88 (dd, *J* = 8.1, 1.9 Hz, 2H, Ar-H), 7.61 – 7.52 (m, 3H, Ar-H), 7.44 (tdd, *J* = 7.1, 5.4, 1.6 Hz, 1H, Ar-H), 7.35 – 7.28 (m, 2H, Ar-H), 2.49 (s, 3H, CH_3); ^{13}C NMR (101 MHz, DMSO) δ 169.78, 161.93, 159.46, 155.21, 147.64, 140.70, 137.41, 131.06, 128.83, 128.48, 126.74, 126.12, 125.38, 124.11, 122.15, 121.78, 116.55, 116.34, 21.01; m/z = 309.42 [M+2H]$^+$;]$^+$; Anal. calcd. for $C_{19}H_{14}FNO_2$: C, 74.26; H, 4.59; F, 6.18; N, 4.56; O,10.41; found: C, 74.65; H, 4.96; N, 4.93.

2 – [2-(3-Methoxyphenyl)vinyl]quinolin-8-ol (**13b**). Method B, a yellow solid, yield 54%, mp 115°C, ^{1}H NMR (400 MHz, DMSO) δ 9.55 (bs, 1H, OH), 8.30 (d, *J* = 8.6 Hz, 1H, Ar-H), 8.10 (d, *J* = 16.2 Hz, 1H, C=C-H), 7.78 (d, *J* = 8.6 Hz, 1H, Ar-H), 7.50 (d, *J* = 16.2 Hz, 1H, C=C-H), 7.40 – 7.34 (m, 3H, Ar-H), 7.32 – 7.28 (m, 2H, Ar-H), 7.10 (dd, *J* = 7.0, 1.8 Hz, 1H, Ar-H), 6.94 (dd, *J* = 8.1, 1.5 Hz, 1H, Ar-H), 3.84 (s, 3H, OCH_3); ^{13}C NMR (101 MHz, DMSO) δ 160.15, 153.82, 153.42, 138.63, 138.42, 136.98, 134.76, 130.37, 128.76, 128.17, 127.47, 121.45, 120.23, 118.04, 115.05, 112.46, 111.59, 55.59; m/z = 302.42 [M+Na+2H]$^+$; Anal. calcd. for $C_{18}H_{15}NO_2$: C, 77.96; H, 5.45; N, 5.05; O, 11.54; found: C, 77.61; H, 4.96; N, 5.26; O, 11.18.

4 – (2-(8-Acetoxyquinolin-2-yl)vinyl)benzene-1,2,3-triyl triacetate (14b). Method A, a white solid, yield 88%, mp 194°C, ^{1}H NMR (400 MHz, DMSO) δ 8.44 (d, *J* = 8.6 Hz, 1H, Ar-H),

7.97 – 7.85 (m, 3H, Ar-H), 7.71 (d, *J* = 16.3 Hz, 1H, C=C-H), 7.62 – 7.49 (m, 3H, Ar-H), 7.36 – 7.31 (m, 1H, Ar-H), 2.47 (s, 3H, CH_3), 2.44 (s, 3H, CH_3), 2.35 (s, 3H, CH_3), 2.31 (s, 3H, CH_3); ^{13}C NMR (101 MHz, DMSO) δ 169.69, 168.60, 168.43, 167.65, 155.10, 147.57, 143.69, 142.04, 140.76, 137.40, 135.70, 131.95, 128.89, 128.61, 126.71, 126.64, 126.16, 124.27, 122.22, 121.94, 121.86, 21.08, 20.85, 20.59, 20.32; m/z = 466.03 $[M+2H]^+$; Anal. calcd. for $C_{25}H_{21}NO_8$: C, 64.79; H, 4.57; N, 3.02; O, 27.62; found: C, 64.87; H, 4.24; N, 3.12; O, 27.78.

2 – [2-(2-Ethoxyphenyl)vinyl]quinolin-8-ol (**15b**). Yield 57.5% of a yellow solid, mp 100°C [1].

2 – [2-(4-Ethoxyphenyl)vinyl]quinolin-8-ol (**16b**). Yield 20.5% of a yellow solid, mp 130°C [1].

2 – [2-(2-Chlorophenyl)vinyl]quinolin-8-ol (**17b**). Yield 91% of a yellow solid, mp 125°C [1].

2 – [2-(2-Hydroxyphenyl)vinyl]quinolin-8-ol (**18b**). Yield 30% of an orange solid, mp 316°C [1].

2 – [2-(3-Hydroxyphenyl)vinyl]quinolin-8-ol (**19b**). Yield 50% of an orange solid, mp 215°C [1].

2 – [2-(2,4-Dihydroxyphenyl)vinyl]quinolin-8-ol (**20b**). Yield 58% of a brick-red solid, mp 273°C [1].

5,7-Dichloro-2 – [2-(2-ethoxyphenyl)vinyl]quinolin-8-ol (**1c**). Yield 26% of a beige crystalline compound, mp 100-102°C [1].

5,7-Dichloro-2 – [2-(2-chlorophenyl)vinyl]quinolin-8-ol (**2c**). Yield 47% of beige crystals, mp 205-210°C [1,3].

5,7-Dichloro-2 – [2-(4-ethoxyphenyl)vinyl]quinolin-8-ol (**3c**). Yield 9% of a beige crystalline compound, mp 156-158°C [1].

Literature

[1] W. Cieslik, R. Musiol, J.E. Nycz, J. Jampilek, M. Vejsova, M. Wolff, et al., Contribution to investigation of antimicrobial activity of styrylquinolines., Bioorg Med Chem. 20 (2012) 6960–8.

[2] B. Machura, M. Wolff, W. Kowalczyk, R. Musiol, Novel rhenium(V) complexes of 8-hydroxyquinoline derivatives – Synthesis, spectroscopic characterization, X-ray structure and DFT calculations, Polyhedron. 33 (2012) 388–395.

[3] B. Machura, M. Wolff, W. Cieślik, R. Musioł, Novel oxorhenium(V) complexes of 8-hydroxyquinoline derivatives – Synthesis, spectroscopic characterization, X-ray crystal structures and DFT calculations, Polyhedron. 51 (2013) 263–274.
