# Supplementary material for: Design, Synthesis and In Vitro Activity of Anticancer Styrylquinolines. The p53 Independent Mechanism of Action
Source: PLoS One. 2015 Nov 23;10(11):e0142678. doi: 10.1371/journal.pone.0142678 (PMC4657899; doi:10.1371/journal.pone.0142678)
Supplement: S1 Table — (PDF) [file pone.0142678.s003.pdf]

S1 Table Fluorescent and absorption properties of tested compounds in DMSO.

No	Absorption λ [nm]	Molar absorption coefficient (ε)·10^4 [$M^{-1}cm^{-1}$]	Emission λ_{max} [nm]	Fluorescence intensity[a]
**1a**	282<br>358	1.83<br>2.11	438	4934
**2a**	358	2.08	438	2441
**3a**	290<br>358	1.49<br>2.43	438	2948
**1b**	304<br>348	3.38<br>1.95	464	2722
**2b**	286<br>346	2.72<br>2.15	404	2095
**3b**	298<br>344	3.94<br>1.96	479	1895
**4b**	302<br>344	4.25<br>2.06	476	1673
**5b**	288<br>348	2.36<br>1.82	409	912
**6b**	294<br>346	3.32<br>2.46	408	1037
**7b**	288<br>350	2.23<br>2.18	410	6168
**8b**	286<br>346	2.92<br>2.36	413	3418
**9b**	372	1.26	475	552
**10b**	284<br>340	2.43<br>1.72	407	1552
**11b**	300<br>342	3.65<br>1.75	478	1633
**12b**	280<br>344	3.16<br>2.18	408	861
**13b**	298<br>348	3.56<br>1.97	468	2184
**14b**	288<br>346	2.58<br>2.22	410	941
**15b**	294<br>354	2.71<br>2.08	462	2483
**16b**	310<br>356	0.89<br>0.61	450	1204
**17b**	288<br>348	2.75<br>2.02	410	1706
**18b**	294<br>358	1.84<br>1.46	450	2400
**19b**	300<br>350	2.53<br>1.48	462	1804
**20b**	376	1.52	474	1592
**1c**	302	1.31	440	192
**2c**	304	3.41	441	117

	348	1.63		
**3c**	302<br>360	2.13<br>1.68	440	212

[a] c = 1.56E-5 M
