# Supplementary material for: Design, Synthesis and In Vitro Activity of Anticancer Styrylquinolines. The p53 Independent Mechanism of Action
Source: PLoS One. 2015 Nov 23;10(11):e0142678. doi: 10.1371/journal.pone.0142678 (PMC4657899; doi:10.1371/journal.pone.0142678)
Supplement: S2 Table — (PDF) [file pone.0142678.s004.pdf]

S2 Table. comparison of antiprofliferativfe activity of the selected compounds measured in metabolic and staining tests.

No	ACTIVITY MTS		TRYPAN BLUE	
	HCT116 +/+	HCT116 -/-	HCT116 +/+	HCT116 -/-
3a	>25	>25	>25	>25
5b	5,13±1,41	2,99±0,61	5,295±2,625	5,359±1,834
6b	9,41±2,17	3,34±0,58	7,312±3,140	12,02±5,436
12b	12,07±3,02	5,78±0,78	9,035±2,185	4,707±1,277
1c	5,93±0,97	3,25±1,81	4,849±1,692	4,338±1,704
2c	1,88±0,85	2,86±1,00	1,068±0,462	3,059±1,186
3c	2,23±0,81	3,53±0,83	3,230±1,984	6,144±2,129
DOX	5,95/1,3	1,65/2,2	2,835±0,761	1,280±0,806

The cells were seeded in 3 cm Petri dishes (Nunc) and incubated at 37°C. After 24h, solutions of 1c, 2c, 3c, 5b, 6b, 12b and 3a were prepared at varying concentrations and were added. After a 72 h incubation with tested compounds, the cells were trypsinized with 0.05% Trypsin-EDTA solution (Sigma). Then, 90µl of suspension cells transferred into 96-well plate containing 10µl Trypan blue reagent in wells. The cells were counted under an optical inverted microscope Olympus CKX31. The results from three independent experiment were calculated as IC_{50} values using GraphPad Prism 5 software.
