# Supplementary material for: Design, Synthesis and In Vitro Activity of Anticancer Styrylquinolines. The p53 Independent Mechanism of Action
Source: PLoS One. 2015 Nov 23;10(11):e0142678. doi: 10.1371/journal.pone.0142678 (PMC4657899; doi:10.1371/journal.pone.0142678)
Supplement: S3 Table — (PDF) [file pone.0142678.s005.pdf]

S3 Table. DNA binding properties of styrylquinolines

Compound	Absorption	Changes in absorbance	% hypochromism	Δε M^{-1} cm^{-1}	shift*
**2c**	300	hypochromism	14,4	2846,7	5
**3c**	390	hypochromism	11,6	557,8	8
**5b**	428	hypochromism	41,3	4797,8	14
**6b**	434	hypochromism	31,0	7335,6	14
**CP-31398**	312; 350	hypochromism	41,4; 37,4	1515,6; 1693,3	0
**DOX**	480	hypochromism	34,2	3235,6	10

*for the wavelengths of maximum absorption for alone and DNA-bound compounds.
